# Epigenetic addition of m^5^C to HBV transcripts promotes viral replication and evasion of innate antiviral responses

**DOI:** 10.1038/s41419-023-06412-9

**Published:** 2024-01-12

**Authors:** Shuang Ding, Haibin Liu, Lijuan Liu, Li Ma, Zhen Chen, Miao Zhu, Lishi Liu, Xueyan Zhang, Haojie Hao, Li Zuo, Jingwen Yang, Xiulin Wu, Ping Zhou, Fang Huang, Fan Zhu, Wuxiang Guan

**Affiliations:** 1 State Key Laboratory of Virology, Department of Medical Microbiology, School of Basic Medical Sciences, Wuhan University, Wuhan, Hubei 430071 China; 2 Center for Emerging Infectious Diseases, Wuhan Institute of Virology, Center for Biosafety Mega-Science, Chinese Academy of Sciences, Wuhan, Hubei 430207 China; 3 Hubei JiangXia Laboratory, Wuhan, Hubei 430200 China; 4 Hubei Province Key Laboratory of Allergy & Immunology, Wuhan University, Wuhan, Hubei 430071 China

**Keywords:** Infection, RNA modification

## Abstract

Eukaryotic five-methylcytosine (m^5^C) is an important regulator of viral RNA splicing, stability, and translation. However, its role in HBV replication remains largely unknown. In this study, functional m^5^C sites are identified in hepatitis B virus (HBV) mRNA. The m^5^C modification at nt 1291 is not only indispensable for Aly/REF export factor (ALYREF) recognition to promote viral mRNA export and HBx translation but also for the inhibition of RIG-I binding to suppress interferon-β (IFN-β) production. Moreover, NOP2/Sun RNA methyltransferase 2 (NSUN2) catalyzes the addition of m^5^C to HBV mRNA and is transcriptionally downregulated by the viral protein HBx, which suppresses the binding of EGR1 to the NSUN2 promoter. Additionally, NSUN2 expression correlates with m^5^C modification of type I IFN mRNA in host cells, thus, positively regulating IFN expression. Hence, the delicate regulation of NSUN2 expression induces m^5^C modification of HBV mRNA while decreasing the levels of m^5^C in host IFN mRNA, making it a vital component of the HBV life cycle. These findings provide new molecular insights into the mechanism of HBV-mediated IFN inhibition and may inform the development of new IFN-α based therapies.

## Introduction

RNA epigenetic modifications exert important effects in myriad biological processes by regulating RNA export [1–3], stability [4–6], and translation [7–9]. The regulatory functions of certain eukaryotic RNA modifications, including N6-methyladenosine (m^6^A), 5-methylcytosine (m^5^C), and N4-acetylcytidine (ac4C), have been well characterized [10, 11]. In particular, m^5^C is formed via the addition of a methyl group from S-adenosylmethionine (SAM) to the carbon-5 position of cytosine in RNA; this process is catalyzed by NOL1/NOP2/Sun domain (NSUN) family members or DNA methyltransferase-2 (DNMT2) [12]. In addition to ribosomal RNAs (rRNAs) and transfer RNAs (tRNAs), m^5^C is present in messenger RNAs (mRNAs) [13]. NOP2/Sun RNA Methyltransferase 2 (NSUN2) functions as a major methyltransferase associated with m^5^C in mRNA. Meanwhile, Aly/REF export factor (ALYREF) and Y-box binding protein 1 (YBX1) serve as “reader” proteins that recognize m^5^C and promote mRNA export from the nucleus, or stabilize RNA, respectively [13–16]. However, the distribution of m^5^C in mRNA varies among cell types. That is, although m^5^C is distributed throughout the mRNA transcript, it may be enriched in specific regions, such as the coding sequence (CDS) [17, 18], 3′ untranslated region (UTR) [18, 19] or the region proximal to the start codon [20–22].

Hepatitis B virus (HBV) infection is a common cause of cirrhosis and cancer. Although the HBV vaccine offers 98%–100% protection against infection, HBV infection remains a global health issue, with significant morbidity and mortality (https://www.who.int/news-room/fact-sheets/detail/hepatitis-b). HBV contains a relaxed-circular DNA genome of approximately 3.2 kb that is transcribed into four HBV mRNA transcripts (3.5 kb, 2.4 kb, 2.1 kb, and 0.7 kb). Among them, the 3.5 kb mRNA encodes three viral proteins, core antigen (HBc), E antigen (HBe), and polymerase (Pol); the 2.4 and 2.1 kb mRNAs encode three S antigens, preS1, preS2, and HBs; whereas the 0.7 kb mRNA encodes X protein (HBx) [23]. HBx is a multi-functional protein that promotes HBV transcription by interacting with damage specific DNA binding protein 1 (DDB1) and recruiting E3 ligase to degrade the structural maintenance of chromosome protein (Smc)5/6 complex [24]. It also interferes with interferon (IFN) production and signaling by targeting mitochondrial antiviral signaling protein (MAVS) [25, 26], cytokine signaling 3 (SOCS3), and protein phosphatase 2 A (PP2A) [27].

Similar to other RNA modifications, m^5^C in viral RNA influences viral replication by regulating RNA splicing, stability, and translation [28]. m^5^C in HIV-1 mRNA is modified by NSUN2, whereas the loss of NSUN2 perturbs HIV-1 alternative splicing and ribosomal recruitment [28]. Meanwhile, m^5^C in the EBV-encoded non-coding RNA, Epstein-Barr virus-encoded protein 1 (EBER1) is essential for viral lytic replication and negatively impacts RNA stability [29]. Despite these few viral studies emphasizing the importance of m^5^C modification during viral infection [28–30], it remains largely unclear how viruses hijack and manipulate the host m^5^C system to promote viral replication.

RNA modification also significantly suppresses RNA-induced innate immune responses in mammalian cells [31]. The most abundant internal RNA modification, m^6^A, serves as a molecular signature that assists viruses, including HBV, in evading the immune system [32–34]. Given that stimulator of IFN gene protein (STING)-mediated DNA sensing is deficient in hepatocytes [35], HBV infection triggers an innate immune response through retinoic acid-inducible protein I (RIG-I)-mediated RNA sensing [36]. RIG-I binds to the 5′ epsilon stem-loop of HBV pre-genomic RNA (pgRNA) to induce type III, not type I, interferon responses [36]. However, Adenosine1907 in the 5’ epsilon stem-loop is typically m^6^A-modified, which reduces the interaction between pgRNA and RIG-I and inhibits IRF-3–mediated IFN production [34]. Meanwhile, m^5^C-modified nucleotides do not trigger RIG-I-mediated immune signaling [37]. However, the function of HBV m^5^C in virus-induced immune evasion has not been elucidated.

In this study, we characterize the role of m^5^C and its methyltransferase NSUN2 in the HBV-induced innate immune response and demonstrate that the precise balance of NSUN2 expression is vital for the HBV life cycle. That is, reduced NSUN2 expression is sufficient for maintaining HBV m^5^C to facilitate efficient viral infection, however, does not increase IFN m^5^C abundance, preventing the induction of IFN expression. Our study reveals that HBV m^5^C and NSUN2 are important factors in HBV-induced innate immune responses.

## Results

### HBV m^5^Cs are essential for viral replication

**Fig. 1:**
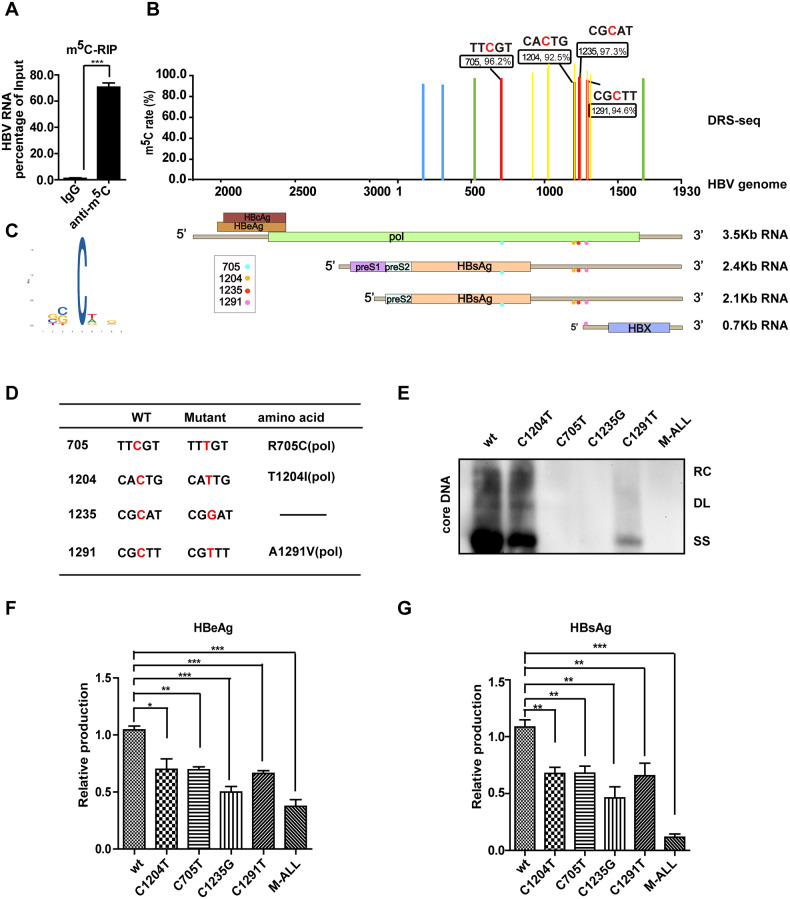
Identification of m^5^C in HBV mRNA essential for viral replication. **A** Total level of HBV m^5^C quantified by m^5^C-RIP. HBV RNA transcripts were immunoprecipitated by m^5^C specific antibody in HepG2.2.15 cells stably transfected with HBV genomic DNA. The enriched HBV RNA was then measured by qPCR with primers targeting the 3′-UTR. **B** Top panel: rate of each m^5^C site, named according to HBV genomic position, quantified by Direct-RNA sequencing with purified polyA+ RNAs from HepG2.2.15 cells or AAV-HBV transduced mouse liver. Identified m^5^C sites with a rate > 90% in two independent experiments with HepG2.2.15 cells are labeled in red, while those with a rate > 90% in one experiment were labeled in green or blue. The m^5^C sites with a rate > 90% identified in AAV-HBV transduced mouse liver were labeled in yellow. Bottom panel: distribution of four confident m^5^C locations (color dots) in HBV RNA transcripts. Four major transcripts from HBV genome with coding potentials are shown. **C** m^5^C motif predicted by MEME [40]. **D**–**G** HBV m^5^Cs are essential for viral replication. The table shows point mutations introduced to each m^5^C and the altered amino acids in proteins. **D** M-ALL mutant containing all m^5^C mutations. Huh7 cells were transfected with HBV 1.1-mer wild-type or mutants. The core-associated DNA was detected by Southern blot using a probe spanning from nt 1 to nt 3182 at 72 h post-transfection. **E** RC relaxed circular DNA, DL duplex-linear DNA, SS single-stranded DNA. Secretion of HBeAg (**F**) and HBsAg (**G**) in cell culture supernatant quantified by ELISA at 72 h post-transfection.

To explore whether m^5^C is present in HBV mRNA, the overall level of m^5^C was determined using m^5^C-methylated RNA immunoprecipitation (m^5^C-RIP) in HepG2.2.15 cells stably transfected with the HBV ayw strain. Approximately 70-fold more HBV mRNA was immunoprecipitated with an m^5^C specific antibody compared with the negative control IgG antibody (Fig. 1A), indicating that HBV mRNA contained m^5^C modifications. The precise m^5^C positions were then determined by nanopore direct RNA sequencing (DRS-seq) using poly (A) + RNAs purified from HepG2.2.15 cells. Four sites, m^5^C-705, m^5^C-1204, m^5^C-1235, and m^5^C-1291, were identified by applying two stringent criteria: (1) the m^5^C rate of each candidate site was at least 90%; (2) the candidate sites could be re-detected by independent DRS (Fig. 1B and Table S1). Consistently, m^5^C-1204 and m^5^C-1291 with m^5^C rate more than 90% were also identified in DRS-seq of poly (A) + RNAs from AAV-HBV transduced mouse liver [38, 39] (Fig. 1B and Table S1). To further confirm the identified HBV m^5^Cs, bisulfite sequencing (BS-seq) was performed on HBV 1.1-mer-transfected cells. Three sites, m^5^C-1204, m^5^C-1235, and m^5^C-1291, were detected using BS-seq (Table S1), however, their detection rates were lower. Statistical m^5^C motif analysis using MEME [40] showed no obvious base preference around the candidate m^5^C sites in either DRS-seq or BS-seq (Fig. 1C).

To investigate the function of HBV m^5^C in the HBV life cycle, point mutations were introduced at four identified m^5^C positions in the HBV genome (Fig. 1D). Three non-synonymous mutations were generated due to limited mutagenesis options (Fig. 1D). The viral DNA replication of mutants was then examined by Southern blot. As shown in Fig. 1E, the synthesis of core-associated DNA including relaxed circular DNA, duplex-linear DNA, and single-stranded DNA was relatively eliminated in the C705T, C1291T, C1235G, and M-ALL mutants, and decreased in the C1204T mutant. In addition, compared to wild-type HBV, all mutants resulted in a significant reduction in HBeAg and HBsAg secretion which was quantified by ELISA in Huh7 cells, while the M-ALL mutant exhibited a synergistic inhibitory effect (Fig. 1F, G). These data indicate that the m^5^Cs are functional and facilitate HBV replication.

### HBV m^5^C-1291 promotes viral RNA export and translation and suppresses RIG-I recognition

**Fig. 2:**
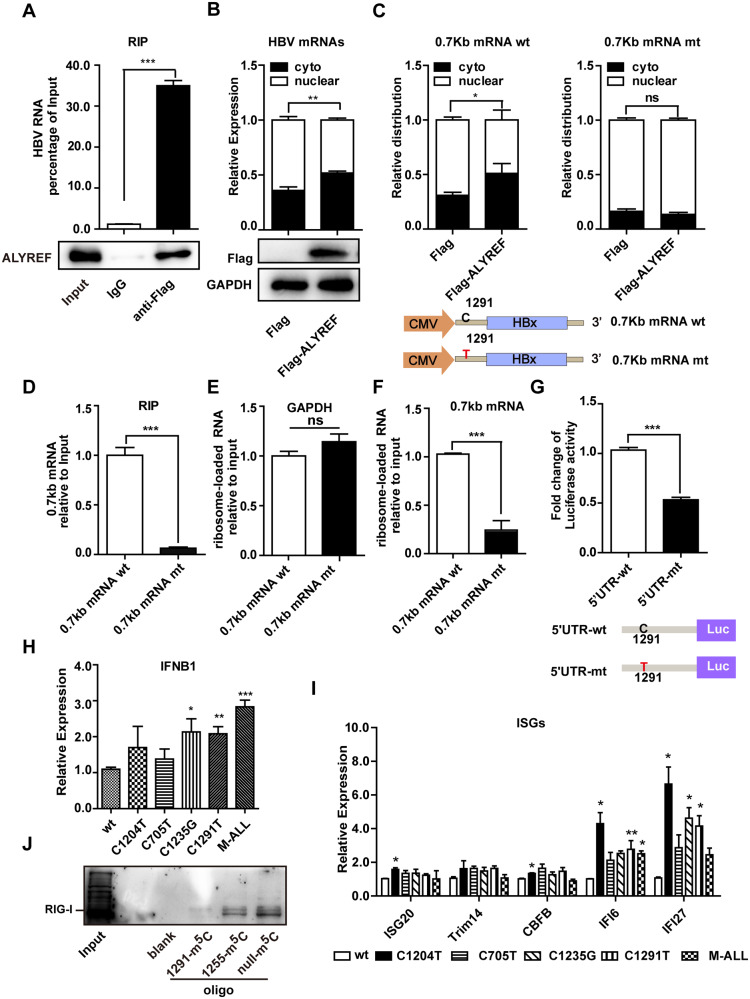
HBV m^5^C-1291 promotes viral RNA export and translation and prevents RIG-I recognition of viral RNA. **A** HBV transcripts are associated with ALYREF. Huh7 cells were transfected with Flag-ALYREF and then with HBV 1.1-mer at 24 h post-transfection. HBV transcripts were immunoprecipitated by anti-Flag antibody at 48 h post-transfection. The enriched HBV RNA was measured by qPCR (**A** top). Immunoprecipitated ALYREF examined by western blot. (**A** bottom). **B** ALYREF promotes HBV RNA export. Subcellular distribution of HBV transcripts in the cytoplasmic and nuclear fractions examined by qPCR after overexpression of ALYREF for 48 h (**B** top panel). ALYREF expression examined by western blot (B, bottom). **C**–**E** The m^5^C-1291 promotes 0.7 kb mRNA export. A point mutation of C1291T is shown, which was introduced to m^5^C-1291 in the cDNA reporter of HBV 0.7 kb mRNA (**C** bottom). Huh7 cells were co-transfected with Flag-ALYREF, and cDNA of 0.7 kb mRNA-wt or 0.7 kb mRNA-mt for 48 h, respectively. Subcellular distribution of 0.7 kb mRNA-wt or 0.7 kb mRNA-mt were examined as described in (**B**) (**C** top). HBV 0.7 kb mRNA wt or mt were then immunoprecipitated by anti-Flag antibody against Flag-ALYREF and measured by qPCR (**D**). **E**–**G** The m^5^C-1291 promotes 0.7 kb mRNA translation. Huh7 cells were transfected with cDNA of 0.7 kb mRNA-wt or 0.7 kb mRNA-mutant for 48 h. Cell lysates were hyper-centrifuged in a sucrose gradient after cycloheximide treatment, and RNA was extracted from the layer with maximum absorbance at OD260. Ribosome-associated HBV 0.7 kb mRNA was assessed by qPCR (**F**). GAPDH was used as a control (**E**). The 5′-UTR of 0.7 kb mRNA-wt or 0.7 kb mRNA-mt were fused to firefly luciferase (Fluc) reporter, respectively (**G** bottom). Huh7 cells were transfected with wild-type or mutant 5′-UTR Fluc reporter. The relative luciferase activities were calculated by dual luciferase assay at 24 h post-transfection (**G** top). Renilla luciferase (Rluc) served as an internal control. (**H**) HBV m^5^C mutants increase IFN-β mRNA expression. Huh7 cells were co-transfected with poly I:C and HBV 1.1-mer wild type or mutants. The expression of IFN-β was assessed by RT-qPCR at 18 h post-transfection. (**I**) Expression of five ISGs assessed by RT-qPCR at 18 h post-transfection. **J** The m^5^C-1291 blocks RIG-I recognition. RNA-oligo pulldown was performed with huh7 cell lysate using biotin-labeled oligos, which contained m^5^C-1291, m^5^C-1255, or non-m^5^C, respectively. The pulled-down proteins were immunoblotted by anti-RIG-I antibody. Pull down without oligos (Beads) served as the control. The bar represents the mean from three independent experiments. ***P < 0.001; **P < 0.01; *P < 0.05.

m^5^C has important roles in RNA export, stability, and translation [13, 15]. The HBV genome primarily encodes at least four mRNAs (Fig. 1B); among the four identified HBV m^5^Cs, only m^5^C-1291 was located in all four mRNAs and was in the 5’-UTR of the 0.7 kb mRNA. Therefore, m^5^C-1291 was selected for further functional analysis. ALYREF is an m^5^C reader that promotes mRNA export [30]. HBV mRNA was co-immunoprecipitated with ALYREF in a formaldehyde-crosslinked RNA immunoprecipitation (RIP) assay, indicating ALYREF bound HBV mRNA (Fig. 2A). Fractionation experiments revealed that ALYREF overexpression significantly increased the export of nuclear HBV mRNA into the cytoplasm (Fig. 2B). The fractionation efficiency was verified by qPCR analysis of glyceraldehyde 3-phosphate dehydrogenase (*GAPDH*) and *U6* (Fig. S1A). To further confirm this result, we constructed a cDNA reporter of 0.7 kb mRNA driven by the CMV promoter according to the reported HBV mRNA isoforms (Fig. 2C) [41]. Notably, the m^5^C-1291 mutation did not affect the stability of the 0.7 kb mRNA after transcription inhibition by Actinomycin D (Fig. S1B). Meanwhile, ALYREF overexpression significantly promoted the export of the wild-type 0.7 kb mRNA, but not that of the mutant 0.7 kb mRNA (Fig. 2C). The binding efficiency of ALYREF to the mutant 0.7 kb mRNA was lower than that of the wild-type 0.7 kb mRNA in the RIP assay (Fig. 2D). In summary, ALYREF binds m^5^C-1291 and promotes the export of HBV mRNA.

The HBV 0.7 kb mRNA potentially encodes the HBx protein. To investigate whether m^5^C-1291 is important for HBx translation, a ribosome-loading assay was performed to determine the association between the HBV 0.7 kb mRNA and ribosomes. GAPDH was used as a negative control (Fig. 2E). Approximately 4-fold more ribosomes were loaded onto wild-type 0.7 kb mRNA compared to mutant 0.7 kb mRNA (Fig. 2F). Furthermore, we inserted the wild-type and mutant 5′-UTR of the 0.7 kb mRNA into a luciferase reporter and evaluated the luciferase activity via dual-luciferase assay (Fig. 2G bottom) [41]. The wild type 5′-UTR exhibited 2-fold higher luciferase activity than the mutant (Fig. 2G top). Taken together, these data suggest that HBV m^5^C-1291 promotes 0.7 kb mRNA translation.

RNA modifications suppress toll-like receptor (TLR) and RIG-I triggered innate immune signaling [31, 37]. Transfection with m^5^C mutants induced upregulation of IFN-β (Fig. 2H and Fig. S1C) and certain ISG mRNAs (Fig. 2I) in poly I:C treated cells by qPCR assays. Similar results were obtained without poly I:C treatment (Fig. S1D, E). RIG-I can be activated by double-stranded RNA in the absence of a 5′-triphosphate terminus [42]; meanwhile, m^5^C-1291 was predicted to be located in the stem region of a folded 5′-UTR structure (nt 1250–1375 in the HBV genome) by RNA structure folding program (http://www.mfold.org/mfold/applications/rna-folding-form.php; ΔG = −36.40; Fig. S1F and Table S2). To explore whether m^5^C-1291 affects RIG-I recognition [36], an RNA oligo pulldown assay with Huh7 cell extract containing endogenous RIG-I was performed using an RNA oligo (nt 1250–1329) derived from the 5′-UTR of the 0.7 kb mRNA. Unmodified RNA oligos exhibited a stronger interaction with RIG-I than m^5^C-modified RNA oligos (Fig. 2J). Collectively, these results indicate that m^5^C-1291 is important for viral RNA to escape RIG-I recognition.

### HBx interacts with EGR1 to downregulate NSUN2 promoter activity

**Fig. 3:**
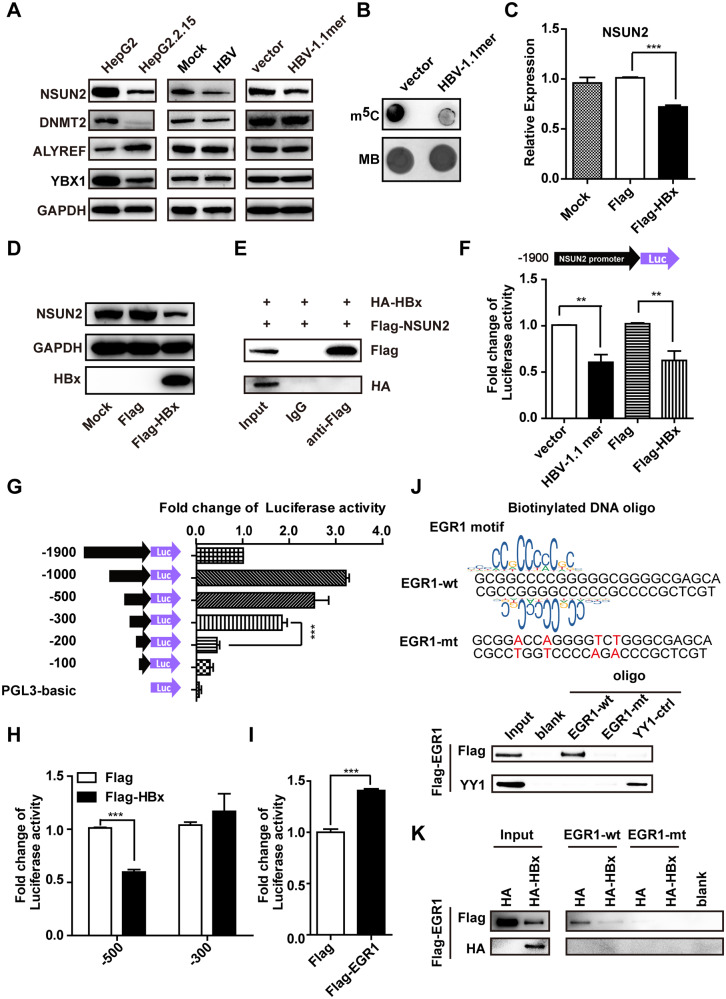
HBV infection downregulates NSUN2 expression through HBx, EGR1, and SP1. **A** HBV infection downregulates NSUN2 expression. The endogenous expression of NSUN2, DNMT2, ALYREF, and YBX1 quantified by western blot in HepG2.2.15 cells or HepG2 cells (**A** left), HepG2-NTCP cells with or without HBV infection for 7 days (A middle), and Huh7 cells transiently transfected with HBV 1.1-mer or control vector for 72 h (**A** right). **B** The global m^5^C levels in Huh7 cells transiently transfected with HBV 1.1-mer or control vector for 72 h were examined by m^5^C dot blot. Methylene blue staining served as the RNA loading control. **C**, **D** Viral protein, HBx is responsible for NSUN2 downregulation by HBV infection. Huh7 cells were transfected with Flag-HBx or flag vector, respectively. Transfection reagent served as the MOCK control. The endogenous RNA level of NSUN2 was quantified by qPCR at 24 h post-transfection. GAPDH was used as an internal control (**C**). The expression of NSUN2 protein and exogenous HBx protein were accessed by western blot, and GAPDH served as the loading control (**D**). **E** Huh7 cells were co-transfected with HA-HBx and Flag-NSUN2. Co-IP with an anti-Flag was performed 48 h post-transfection. HBx protein was immunoblotted with an anti-HA. **F** HBV transfection or HBx expression suppresses NSUN2 promoter activity. A 2-kb DNA fragment (−1900 to +100) of the NSUN2 promoter was fused to Firefly luciferase (Fluc) reporter. Fluc reporter was co-transfected with the control vector, HBV 1.1-mer, flag vector, or Flag-HBx in Huh7 cells. The promoter activity was determined by dual luciferase assay 24 h post-transfection. Renilla luciferase (Rluc) served as a promoter control. **G** Mapping of the core promoter by 5′-end deletions in the Fluc reporter. The Huh7 cells were transfected with truncated Fluc reporter. The promoter activity was determined by dual luciferase assay at 24 h post-transfection. (**H**) Identification of HBx response region in the NSUN2 promoter. Two truncated Fluc reporters, −500 or −300 were co-transfected with Flag-HBx or flag vector in Huh7 cells. The promoter activity was determined by dual luciferase assay as above. **I** Fluc reporter was co-transfected with Flag vector or Flag-EGR1 in Huh7 cells. The promoter activity was determined by dual luciferase assay 24 h post-transfection. **J** Biotinylated double-stranded WT or MT oligos contained EGR1 or mutated motif, respectively. The pulled-down EGR1 protein by WT or MT oligos was accessed by western blot. The oligo containing YY1 motif served as the positive control. **K** Oligo pulled down was performed using cell lysates from Huh7 cells transfected with Flag-HBx or flag vector, respectively. EGR1 protein was accessed by western blot. The bar represents the mean from three independent experiments. ***P < 0.001; **P < 0.01; *P < 0.05.

HBV does not encode methyltransferases. Thus, the expression of host m^5^C “writers,” NSUN2 and DNMT2, and “readers,” ALYREF and YBX1 were analyzed by Western blot [13, 15, 16, 43, 44]. A decrease in NSUN2, DNMT2, and YBX1, and an increase in ALYREF were observed in HepG2.2.15 cells compared with parent HepG2 cells (Fig. 3A, left). The decrease in NSUN2 was not due to stable HBV expression from DNA integration in HepG2.2.15 cells, since this was also detected in HepG2-NTCP cells infected with HBV for 10 days (Fig. 3A middle and Fig. S2A), and Huh7 cells transiently transfected with HBV 1.1-mer for 72 h (Fig. 3A right). The global m^5^C levels in Huh7 cells was also significantly reduced by HBV-1.1mer transfection. (Fig. 3B). Low NSUN2 expression was consistently observed in HBV patient samples with acute liver failure [45] (Fig. S2B), indicating that HBV infection reduces NSUN2 expression in both cell culture and clinical samples.

To investigate which viral protein contributes NSUN2 downregulation, HBs HBc, and HBx were overexpressed in Huh7 cells, and endogenous NSUN2 expression was analyzed. HBx overexpression reduced NSUN2 mRNA (Fig. 3C and Fig. S2C, D) and protein levels (Fig. 3D). Notably, HBx did not interact with NSUN2 in the co-immunoprecipitation (co-IP) assay (Fig. 3E) or affect the stability of NSUN2 mRNA in the RNA stability assay (Fig. S2E).

**Fig. 4:**
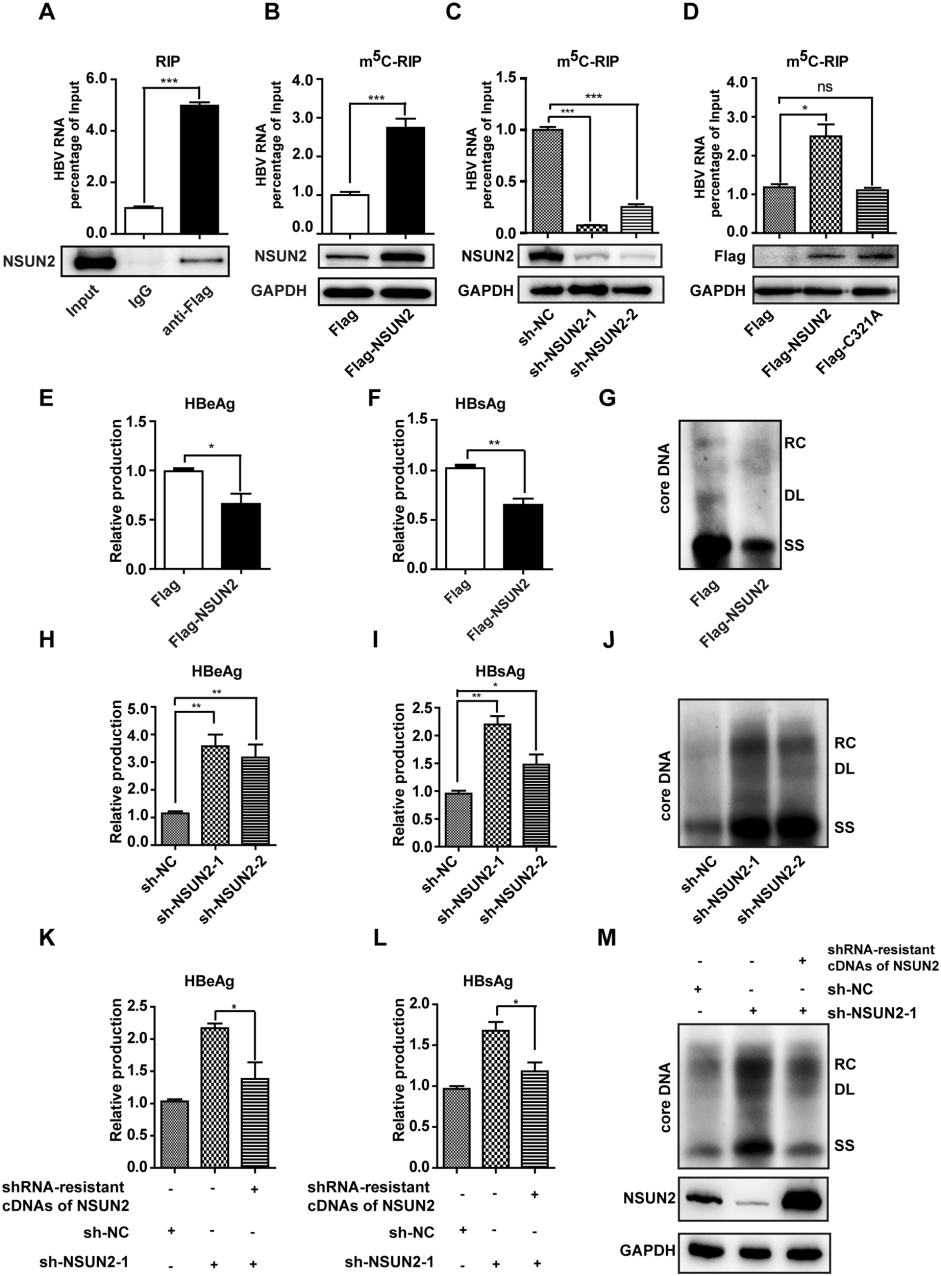
NSUN2 is the methyltransferase of HBV m^5^C and suppresses HBV infection. **A**–**D** NSUN2 serves as the methyltransferase of HBV m^5^C. **A** NSUN2 was associated with HBV mRNA. Huh7 cells were transfected with Flag-NSUN2 and HBV-1.1mer. HBV mRNA were then immunoprecipitated by Flag antibody and measured by qPCR at 48 h post-transfection (**A** top). The immunoprecipitated NSUN2 was examined by western blot (**A** bottom). Overexpression of NSUN2 enhanced the total level of HBV m^5^C determined by m^5^C-RIP (**B**). Knock-down of NSUN2 by two specific shRNAs decreased the total level of HBV m^5^C determined by m^5^C-RIP (**C**). Mutant NSUN2 (C321A) could not increase the level of HBV m^5^C modification compared to wild-type NSUN2 (D). **E**–**G** Overexpression of NSUN2 suppressed HBV antigen secretion and replication. Transfection with flag-NSUN2 and HBV 1.1-mer in Huh7 cells were performed as above. Secretion of viral proteins, HBeAg (**E**) and HBsAg (**F**), in cell culture supernatant were quantified by ELISA at 72 h post-transfection. The core-associated DNA was detected by Southern blot using a probe spanning nt 1 to nt 3182 at 72 h post-transfection (**G**). RC relaxed circular DNA; DL duplex-linear DNA; SS single-stranded DNA. **H**–**J** Knock-down of NSUN2 promotes HBV replication and antigen secretion. NSUN2 was knocked down by two shRNAs as described above. The secretion of HBeAg (**H**) and HBsAg (**I**), and the core-associated DNA (**J**), were examined. (**K**–**M**) Restoration of NSUN2 in NSUN2 knock-down cells reduces the enhanced HBV infection. Huh7 cells expressing NSUN2 shRNAs were transfected with shRNA-resistant NSUN2 cDNA by introducing mutations at shRNA seed regions. The secretion of HBeAg (K) and HBsAg (**L**), and the core-associated DNA (**M**), were examined as above. ***P < 0.001; **P < 0.01; *P < 0.05.

Considering that HBx is a well-studied transcriptional regulator of HBV infection [46], we hypothesized that NSUN2 expression is transcriptionally regulated by HBx. To test this hypothesis, a ~2 kb DNA fragment containing the whole promoter of NSUN2 was inserted into luciferase reporter and the luciferase activities were evaluated using a dual-luciferase assay. Promoter activity was reduced by HBV 1.1-mer transfection or HBx expression (Fig. 3F). To explore the core promoter and cis-regulatory elements, promoter activities of the truncated sequence were tested. Although the −500 and −300 promoters exhibited similar activities (Fig. 3G), only the-500 promoter responded to HBx expression (Fig. 3H). Presumably, the promoter region between −500 nt and −300 nt contained HBx response elements. Moreover, based on our JASPAR database (https://jaspar.genereg.net/) analysis, EGR1, SP1, and ZNF148 may bind to two GC-enriched motifs (−476 to −463 and −350 to −333) in the NSUN2 promoter (Table S3). EGR1, which interacts with HBx [47], was identified as a transactivator of the NSUN2 promoter (Fig. 3I). EGR1 bound to the second GC-enriched motif via DNA oligo pulldown (Fig. 4J), while the expression and binding of EGR1 were decreased by HBx (Fig. 4K). Taken together, these data suggest that HBx suppresses NSUN2 promoter activity by interacting with EGR1 and preventing its binding to the NSUN2 promoter.

### NSUN2 functions as the methyltransferase of HBV m^5^C while suppressing HBV infection

To explore which methyltransferase is responsible for the addition of m^5^C to HBV mRNAs, an RIP assay was performed to determine whether HBV mRNAs are associated with NSUN2 or DNMT2. HBV mRNAs co-immunoprecipitated with NSUN2, not DNMT2 (Fig. 4A and S3A). The overall level of m^5^C in HBV mRNA was increased by *NSUN2* overexpression (Fig. 4B) and decreased by *NSUN2* knockdown in m^5^C-RIP assay (Fig. 4C). However, overexpression or knockdown of *DNMT2* did not impact m^5^C levels in HBV mRNA (Fig. S3B, C). Moreover, the NSUN2 C321A mutant, which disrupts the transient covalent bond to cytosine [28], failed to enhance HBV m^5^C levels compared with wild-type NSUN2 in m^5^C-RIP assay (Fig. 4D). In contrast to cytoplasmic replicating viruses [48], nuclear localization of NSUN2 was not altered by transient HBV transfection (Fig. S3D) or stable HBV ayw strain transfection (Fig. S3E). Thus, NSUN2 directly binds to HBV mRNA to catalyze cytosine methylation in hepatocyte nuclei.

Given that host RNA modification enzymes promote viral replication by modifying viral RNAs [28, 49–51], the role of NSUN2 in the HBV life cycle was investigated. A significant reduction in HBeAg and HBsAg secretion was observed in cells overexpressing *NSUN2* (Fig. 4E, F). Core-associated DNA, including relaxed circular DNA, duplex-linear DNA, and single-stranded DNA, were suppressed by NSUN2 (Fig. 4G). Knockdown of endogenous NSUN2 using two shRNAs enhanced antigen secretion (Fig. 4H, I) and viral replication (Fig. 4J). Restoration of NSUN2 expression via transfection with shRNA-resistant cDNA reversed the enhanced antigen secretion (Fig. 4K, L) and viral replication (Fig. 4M). These data suggest that NSUN2 catalyzes HBV m^5^C addition, however, suppresses HBV replication.

### The delicate balance of NSUN2 expression inhibits IFN production

**Fig. 5:**
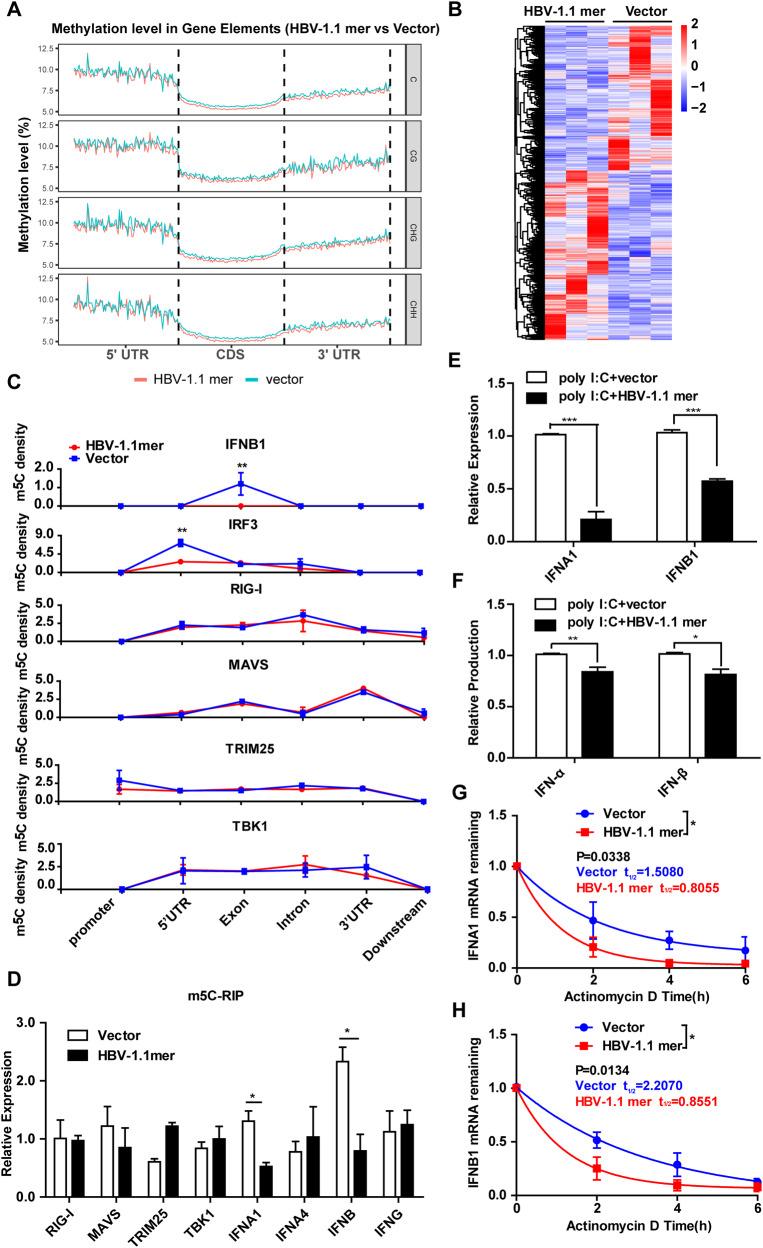
HBV infection decreases m^5^C level in IFN-related genes. **A**–**C** BS-seq was performed with total RNA isolated from Huh7 cells transfected with HBV 1.1-mer at 18 h post-transfection. The average m^5^C density in each gene structure, 5′-UTR, CDS and 3′-UTR was calculated in HBV 1.1-mer transfected or un-transfected cells (**A**). The heatmap shows the differentially methylated region identified in six samples (B). The line graph shows the m^5^C density in each gene structure, promoter, 5′-UTR, exon, intron, 3′-UTR and downstream of several genes related to RIG-I signaling (RIG-I, MAVS, TRIM25, IRF3, and IFNB1) (**C**). **D** validation of the m^5^C level of genes related to RIG-I signaling by m^5^C-RIP assay in HBV 1.1-mer transfected or un-transfected cells. **E**–**H** HBV 1.1-mer transfection inhibits poly I:C induced IFN-α and IFN-β production. Huh7 cells were transfected with poly I:C and HBV 1.1-mer, or poly I:C and control vector for 18 h, respectively. The production (**E**) and mRNA expression (**F**) of IFN-α and IFN-β were assessed by qPCR or ELISA, respectively. The RNA stability of IFNA1 (**G**) and IFNB1 (**H**) RNA was reduced by HBV-1.1 mer transfection. ***P < 0.001; **P < 0.01; *P < 0.05.

To investigate the mechanism by which NSUN2 suppresses HBV replication, altered global m^5^C levels in host genes in HBV 1.1-mer-transfected cells were examined. We performed BS-Seq to analyze differentially methylated cellular RNAs. The sequencing data showed that the m^5^C modifications were primarily distributed in the 5′-UTR (Fig. 5A). Consistent with the globally reduced m^5^C levels (Fig. 3B), the average m^5^C density in CDS and 3′-UTR decreased following HBV 1.1-mer transfection (Fig. 5A and Table S4). A total of 20,148 differentially methylated regions (DMRs) were identified (Table S5), which were hierarchically clustered in a heat map (Fig. 5B). Moreover, the m^5^C levels were significantly altered in cellular RNA involved in innate immune responses, including those encoding IRF3, IFNB1, IFNAR1, IFNAR2, IFNGR2, and IFNGR1 (Table S5).

Given that the HBV-induced immune response correlates with RIG-I recognition [36], we assessed the m^5^C densities of molecules related to RIG-I signaling, including RIG-I, MAVS, TRIM25, TBK1, IRF3, IFNA1, IFNA4, IFNB1, and IFNG. Notably, the m^5^C density in the 5′-UTR of IRF3 and the exon of IFNB1 was significantly decreased (Fig. 5C). Additionally, according to the m^5^C-RIP analysis, m^5^C levels were decreased in IFNA1 and IFNB1 RNAs (Fig. 5D). In addition, HBV 1.1-mer transfection inhibited poly I: C-induced IFNA1 and IFNB1 mRNA in qPCR assays (Fig.5E) and protein secretion in ELISA (Fig. 5F), which was associated with reduced RNA stability (Fig. 5G, H). These data imply that HBV transfection suppresses type I IFN production by reducing m^5^C levels and RNA stability.

**Fig. 6:**
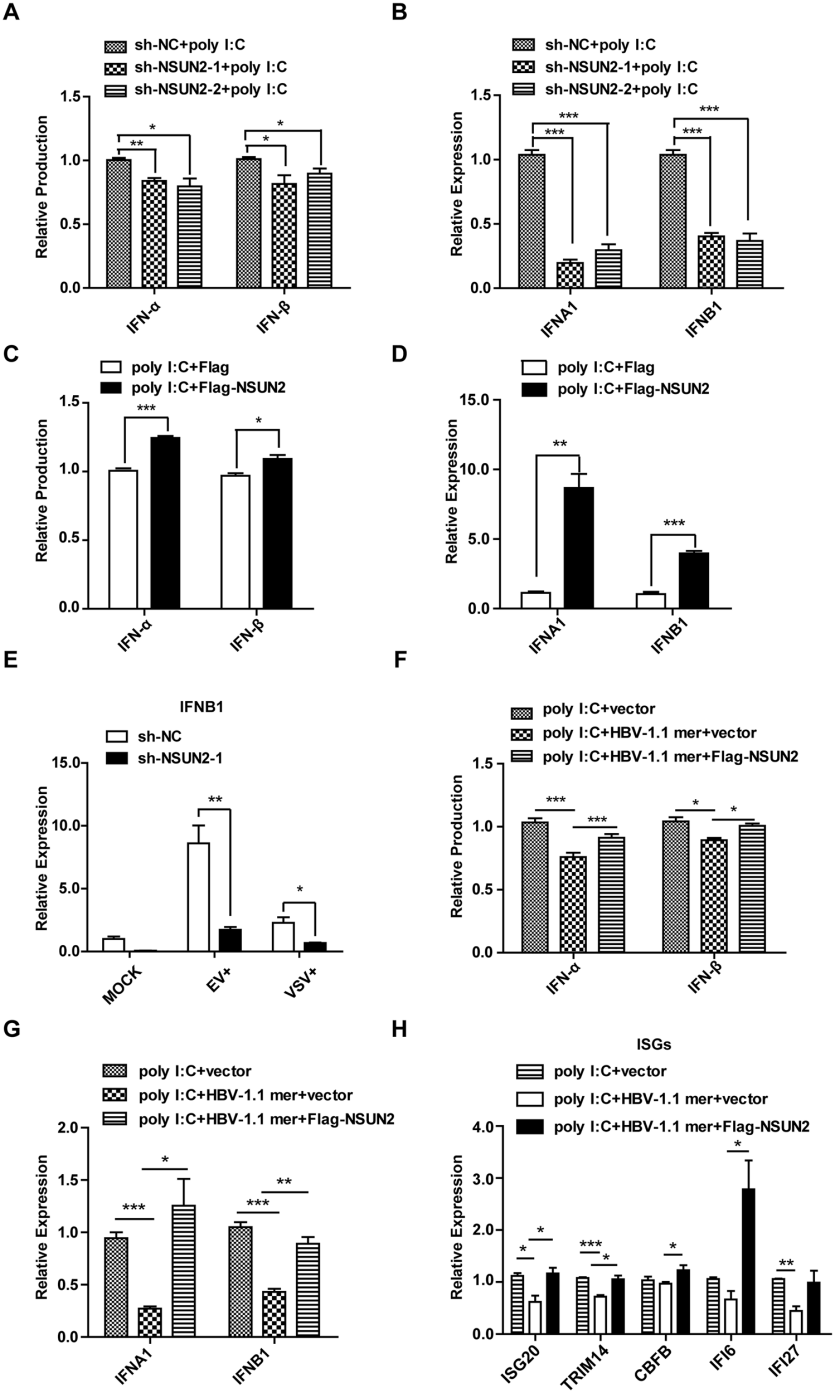
NSUN2 is a positive regulator in poly I:C-induced IFN-α and IFN-β production. **A**, **B** Knock-down of NSUN2 decreases poly I:C-induced IFN-α and IFN-β production. The NSNUN2 or NC shRNA stably expressing cells were treated with poly I:C for 18 h. The protein (**A**) and mRNA expression (**B**) of IFN-α and IFN-β were assessed by ELISA or qPCR, respectively. **C**, **D** Overexpression of NSUN2 increases poly I:C-induced IFN-α and IFN-β production. Huh7 cells were transfected with poly I:C and Flag-NSUN2, or poly I:C and flag vector for 18 h. The protein (**C**) and mRNA expression (**D**) of IFN-α and IFN-β were assessed. **E** NSUN2 positively regulates IFN-β expression in uninfected, EV71-infected, and VSV-infected cells. Huh7 cells were infected with EV71 or VSV, or without virus for 24 h, and IFN-β mRNA expression was assessed. **F**–**H** HBV-mediated IFN inhibition is dependent on NSUN2. Poly I:C was co-transfected with the control vector, HBV 1.1-mer, and flag vector, or HBV 1.1-mer and Flag-NSUN2 in Huh7 cells for 18 h. The protein (**F**) and mRNA expression (**G**) of IFN-α and IFN-β were assessed. The expression of several ISGs was accessed by qPCR (**H**). ***P < 0.001; **P < 0.01; *P < 0.05.

Negligible levels of type I IFN have been detected in patients with HBV; however, the mechanism associated with its downregulation is poorly understood [52]. To explore the correlation between NSUN2 and inhibited IFN production, NSUN2 was depleted or overexpressed, followed by measurement of IFN production by ELISA and IFN expression by qPCR assay. As expected, NSUN2 knockdown decreased the poly I: C-induced IFN-α and IFN-α production (Fig. 6A, B). In contrast, overexpression of NSUN2 increased IFN-α and IFN-β production (Fig. 6C, D). NSUN2 depletion also significantly decreased virus-induced IFN production (Fig. 6E). Restoration of NSUN2 in HBV 1.1-mer-transfected cells rescued the inhibited IFN-α and IFN-β production (Fig. 6F, G), as well as the reduced ISGs (ISG20, TRIM14, CBFB, IFI6 and IFI27; Fig. 6H). Taken together, IFN-α and IFN-β production is closely correlated to the m^5^C levels of their mRNA and NSUN2 expression.

## Discussion

**Fig. 7:**
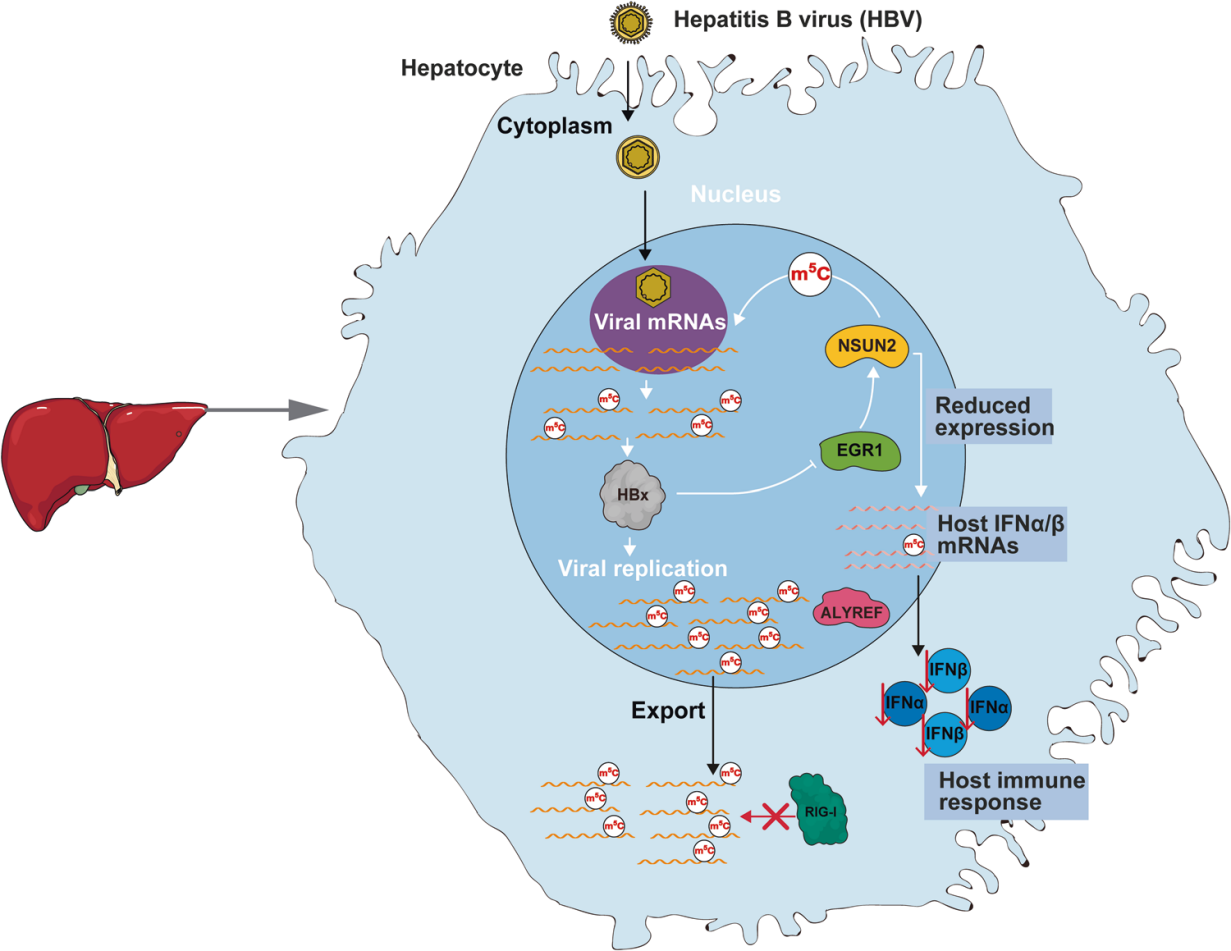
Proposed model of how HBV regulates the host RNA methyltransferase NSUN2 to facilitate its replication. During the HBV infection, NSUN2 is transcriptionally repressed by HBx through inhibition of EGR1 binding to its promoter in the nucleus. The reduced NSUN2 catalyzes m^5^C modifications in HBV mRNAs to promote viral RNA export. However, the m^5^C modifications in the RNAs of IFN-α and IFN-β were decreased due to the repressed level of NSUN2. After the viral and cellular mRNAs are delivered into cytosol, HBV m^5^Cs promote viral RNA translation and inhibit RIG-I recognition. The reduced m^5^Cs in IFN-α and IFN-β RNAs result in low expression of IFN-α and IFN-β, which ultimately leads to efficient HBV replication.

Host RNA modification enzymes are reportedly essential for viral replication and pathogenesis by modifying viral RNA [28, 49–51]. In the present study, we found that NSUN2 plays an important role in the HBV life cycle (Fig. 7). NSUN2 expression is downregulated by HBx which decreases the binding of EGR1 to the NSUN2 promoter. NSUN2 catalyzes the addition of m^5^Cs in HBV mRNAs, and functional m^5^Cs promote HBV RNA export and translation while inhibiting the innate immune response by decreasing RIG-I binding. In contrast, decreased NUSN2 expression reduces m^5^Cs in IFN RNA, resulting in decreased IFN production. Thus, maintaining a delicate balance in NSUN2 expression during HBV infection is important for the viral life cycle.

RNA modifications, including m^6^A, m^5^C, and ac4C, of viral RNA are indispensable for viral replication as they regulate viral RNA splicing, stability, and translation [28, 51]. Certain functional m^5^Cs were identified in HBV mRNA by DRS-seq and confirmed by BS-seq. These m^5^Cs have multiple roles in the HBV lifecycle. First, introducing mutations in HBV m^5^Cs abolishes viral replication and decreases antigen secretion. Second, HBV m^5^C modifications promote viral mRNA export by binding to ALYREF [53]. Third, m^5^C-1291 in the 5′-UTR of the 0.7 kb mRNA promotes HBx translation, which further promotes HBV transcription and influences IFN production [24–27]. Finally, m^5^C-1291 attenuates the immune response by decreasing RIG-I binding to the 0.7 kb HBV mRNA. Owing to the importance of this single nucleotide in HBV infection, a specific targeting strategy combined with HBV therapy may improve the efficiency of HBV infection clearance.

Host RNA modification enzymes are often elevated and hijacked by viral proteins to promote the infection [48, 50, 54, 55]. Depletion of the methyltransferase of m^6^A modification, METTL3, suppresses the production of EV71 [55] and SARS-CoV-2 [48] by disrupting viral RNA stability and translation. However, the expression of NSUN2 was downregulated in HBV-infected cell cultures and patient samples by HBx. To understand the cause of this discrepancy, genome-wide m^5^C levels in cellular RNAs were investigated in HBV DNA-transfected cells. HBV infection led to reduced m^5^C in IFN RNA and decreased IFN secretion, likely mediated by reduced NSUN2 expression. Thus, a balance in NSUN2 expression results in decreased m^5^C levels in IFN RNA, which appears necessary for the virus to complete its life cycle.

NSUN2 expression was moderately downregulated by viral encoded HBx through inhibiting EGR1 binding to NSUN2 promoter. NSUN2 catalyzed the m^5^C addition to both HBV and IFN mRNAs. The repressed NSUN2 expression decreased m^5^C levels and RNA stabilities of IFN genes, which led to impair the immune responses; while the existing amount of NSUN2 was sufficient to catalyze the m^5^C addition in HBV mRNA, which promoted viral RNA exportation and translational efficiency but inhibited the RIG-I recognition of viral RNAs. Thus, we speculated the delicate balance in NSUN2 expression was important during HBV replication.

In previous reports, increased methyltransferases were found to be post-translationally regulated by viral proteins [48, 51, 55]. However, our data revealed a different mechanism of NSUN2 down-regulation during HBV infection, which was transcriptionally suppressed by HBx. We also identified an HBx response region in the NSUN2 promoter. Given that HBx is not a DNA-binding protein, interaction with other host factors or activation of signal transduction is necessary for HBx to target the NSUN2 promoter [56]. In fact, DNA-binding proteins, including DDB1 [24], CBFβ [57], p53 [58] and TFIIB [59] interact with HBx. HBx also physically interacts with EGR1, EGR2, and EGR3, but only transactivates EGR2 and EGR3, not EGR1, by recruiting the coactivator CBP [60]. In this study, we found that EGR1 transactivates the *NUSN2* promoter by binding to the second GC-rich motif while HBx interacts with EGR1 and inhibits its binding, resulting in suppressed *NSUN2* transcription.

IFN production and signaling pathways are compromised by HBV infection [61–64], which may account for the failure of IFN-α therapy in many patients with chronic HBV infection [65]. Here we demonstrated that NUSN2 positively regulates the expression of IFN-α and IFN-β production in HBV-infected cells. Thus, we hypothesize that HBV-mediated IFN inhibition occurs through the downregulation of NSUN2. In fact, restoring NSUN2 expression in HBV cell cultures rescued the inhibition of IFN production. However, the opposite function was recently reported for NSUN2; Zhang et.al. found that NSUN2 depletion transcriptionally upregulates polymerase (Pol) III-transcribed ncRNAs, which serve as RIG-I substrates to induce an IFN response in A549 cells. However, the mechanism of NSUN2-mediated transcriptional regulation is unclear [66]. Considering that hepatocytes were used in the current study, we hypothesize that NUSN2-mediated innate immune responses differ in a cell context-dependent manner, however, this requires further confirmation.

Although our data suggest that m^5^C is preferentially localized in the common region of the four HBV mRNAs, the sequencing reads in our current DRS were not sufficiently long to map all HBV transcripts. Accordingly, we were unable to distinguish transcript-specific m^5^Cs. Moreover, a previous report found that m^5^C modifications generated by NSUN2 are typically localized in GC-rich regions of mRNAs [14], but no preferential sequences were identified around the m^5^C sites by our motif analysis. The motifs of NSUN2-mediated m^5^C modifications differ between ncRNAs and tRNA [67, 68]. Bohnsack et. al. reviewed the motif of NSUN2-mediated m^5^C modifications and proposed that NSUN2 targets the secondary structure of RNA substrates rather than a specific RNA sequence [22]. This hypothesis is supported by the notion that NSUN2-mediated m^5^C_34_ is dependent on the elongated anticodon stem structure of pre-tRNALeu [68]. Although this study provides valuable insights into the role of m^5^C and NSUN2 in HBV pathogenesis, their precise functions require further investigation in animal models and clinical patient cases [69–71].

In summary, m^5^Cs and the host methyltransferase NSUN2 are key factors in HBV-mediated IFN inhibition, indicating that they are promising targets for the development or improvement of HBV therapy.

## Materials and methods

### Cell culture and transfection

Huh7, HepG2, HepG2-NTCP, and HepG2.2.15 cells, which stably express HBV, were cultured in Dulbecco’s modified Eagle’s medium supplemented with 10% fetal bovine serum at 37 °C in a 5% CO_2_ environment. Plasmid transfection was carried out using Lipofectamine 2000 reagent (Invitrogen, Carlsbad, CA, USA; catalog no. 11668-019) according to the manufacturer’s instructions.

### Nanopore direct RNA sequencing (DRS-seq)

PolyA+ RNA was purified from 1 mg of total RNA from HepG2.2.15 cells or AAV-HBV transduced mouse liver [38, 39] using a GenEluteTM mRNA Miniprep Kit (Sigma-Aldrich). Independent experiments of DRS-seq were performed according to the manufacturer’s instructions (Oxford Nanopore DRS protocol; SQKRNA002). Briefly, the sequencing library prepared from PolyA+ RNA was loaded onto the FLO-MIN106D flow cell, followed by a 72 h sequencing run on a MinION device (Oxford Nanopore Technologies). Multi-fast5 reads were base called guppy (v3.1.5) and then converted to single-read fast5 using the command multi_to_single_fast5 in the Oxford Nanopore Technologies API, ont_fast5_api (v3.0.2). Single-fast5 data were mapped to the reference genome of HBV (NC_003977.2) using Tombo (v1.5.1). The specific modifications were detected by the Tombo detect_modifications command, and the all-context alternate model was used to identify 5-methylcytosine in any sequence by running “-alternate-bases 5mC.” Subsequently, the “text_output” command was applied to obtain the methylation score and coverage rate at each position.

### Bisulfite sequencing (BS-seq)

Total RNA was extracted from Huh7 cells transfected with Poly I:C and HBV 1.1-mer at 18 h post-transfection using TRIzol™ Reagent (Thermo Fisher Scientific). PolyA+ RNA was then selected and the library was constructed using the EZ RNA Methylation™ Kit (ZYMO RESEARCH) and KC UMI RNA Library Kit (Wuhan SeqHealth Tech) according to the manufacturer’s instructions. Sequencing of three samples in each condition was performed using a MGISEQ-T7 sequencing system (BGI). Sequencing data was uploaded to the GEO repository (GSE246879). Raw reads were first processed using fastp (version 0.23.0) to remove residual adaptor sequences and low-quality reads [72]. The clean reads were then mapped to the reference genome, and duplicated reads were removed using Bismark (version 0.22.3) [73]. The chromosome depth and coverage were calculated using MosDepth (version 0.3.1) and RSeQC (version 4.0.0) [74]. Methylation detection along the whole genome was conducted using Bismark, which calculates the proportion of methylated reads at each genomic site. DMRs between different groups were detected using methylene (version 0.2–8) [75].

### Plasmid constructs

NSUN2, DNMT2, ALYREF, HBx, HBsAg, and HBcAg expression plasmids were cloned into the PXJ40-Flag/HA vector. The Flag-C321A plasmid was constructed as described previously [28]. shRNA-resistant cDNAs of NSUN2 plasmids were constructed by introducing four site mutations into the NSUN2 expression plasmids. C1204T, C705T, C1235G, C1291T, and M-ALL plasmids were generated by introducing site mutations into the pch9-3091 plasmid. The HBV 0.7 kb mRNA wt and mt plasmids were generated by inserting the 5′-UTR, CDS and 3′-UTR region of HBx from the pch9-3091 plasmid into the PXJ40-HA vector. The 5′-UTR-wt and 5′-UTR-mt plasmids were generated by inserting the HBx wt 5′-UTR and m5C mutant 5′-UTR into the PGL3-basic plasmid. The NSUN2 promoter luciferase reporter plasmid and truncated plasmids were constructed by inserting the −190 to +100, −1000 to +100, −500 to +100, −300 to +100, −200 to +100, and-100 to +100 promoter regions of the NSUN2 genome from huh7 cells into the vector PGL3-basic.

### Dot blot

Ten micrograms of total RNA isolated from huh7 cells were denatured at 75 °C for 5 min and then added dropwise onto the Hybound N+ membrane (GE Healthcare). The membrane was then dried and exposed to UV light to crosslink the RNA samples. The membrane was blocked in 5% skimmed milk and incubated with primary antibody against m^5^C modification (cat. no. ab10805; Abcam, Cambridge, UK) overnight at 4 °C. After three washes, the membranes were incubated with secondary antibodies [horseradish peroxidase (HRP) goat anti-mouse IgG (H + L) 115-035-003] for 1 h at room temperature. Next, 1 mL of the prepared chemiluminescent reagent mix was added to the membrane after three washes. Immunoblot signals were detected using the Tanon-5200 ChemiDoc MP imaging system (Tanon Science & Technology, Shanghai, China). Three independent experiments of Dot blots were performed, and one representative result was shown Fig. 3B.

### Western blotting

Cells were lysed on ice for 30 min with lysis buffer (P0013, Beyotime), centrifuged at 14,000 rpm for 10 min, and denatured at 100 °C. Proteins were separated by SDS-PAGE and transferred to nitrocellulose membranes. The following steps were performed as described previously [51]. The primary antibodies included Flag (cat. no. F1804-1 MG; Sigma-Aldrich), GAPDH (cat. no. 60004-1-lg; Proteintech, Rosemont, IL, USA), NSUN2 (cat. no. 20854-1-AP; Proteintech, Rosemont, IL, USA), DNMT2 (cat. no. 19221-1-AP; Proteintech, Rosemont, IL, USA), ALYREF (cat. no. 16690-1-AP; Proteintech, Rosemont, IL, USA), HA (cat. no. 51064-2-AP; Proteintech, Rosemont, IL, USA), and PreS2 (cat. No. sc-23944; Santa Cruz Biotechnology, Dallas, TX, USA). The anti-HBsAg antibody was gifted by Dr. Bing Yan from the Wuhan Institute of Virology, CAS. At least three independent experiments for each western blot were performed. Original western blots are shown in Supplementary Material.

### Immunofluorescence

Cells were seeded in microplates at 50% confluence and then transfected with the HBV 1.1-mer plasmid, pch9-3091, as described above. The following day, cells were fixed with 3.7% paraformaldehyde for 30 min at room temperature 72 h post-transfection and then permeabilized in 0.5% Triton-X100 on ice for 10 min. After blocking in 3% BSA for 1 h, the cells were incubated with primary antibodies at a dilution of 1:50 for 1 h at room temperature and then incubated with secondary antibodies (A-11001 or A-11011, Invitrogen) for 1 h at room temperature. Finally, the samples were stained with Hoechst for 5 min. Images were obtained using a VoX confocal microscope (PerkinElmer, Waltham, MA, USA).

### M^5^C-methylated RNA immunoprecipitation (m^5^C-RIP) and RT-qPCR

Total RNA was extracted from HepG2.2.15 cells using TRIzol reagent (Invitrogen, Carlsbad, CA, USA). m^5^C-RIP was performed as previously described [55]. Briefly, 300 μg of RNA were resuspend in immunoprecipitation (IP) buffer (150 mmol/L NaCl, 0.1% NP-40, 10 mmol/L Tris-HCl pH 7.4) and incubated with an anti-m^5^C antibody (ab10805, Abcam) or normal rabbit/mouse IgG antibody (Proteintech) overnight at 4 °C. The RNA and antibody mixture was then incubated with 35 μL of magnetic beads (New England Biolabs) for 2 h at 4 °C. Beads were washed with IP buffer six times and then incubated with 300 μL of elution buffer (5 mmol/L Tris-HCl pH 7.5, 1 mmol/L EDTA pH 8.0, 0.05% SDS, 4.2 μL 20 mg/mL proteinase K) at 50 °C for 1.5 h. Eluted RNA was purified using phenol/chloroform. Immunoprecipitated RNA was used for cDNA synthesis using the HiScript 1st Strand cDNA Synthesis Kit (Vazyme) according to the manufacturer’s protocol. The relative RNA level was measured by quantitative PCR (qPCR) using Hieff® qPCR SYBR® Green Master Mix (Yeasen Biotech Co., Shanghai, China) on a CFX Connect real-time system (Bio-Rad Laboratories, Hercules, CA, USA). The primers used for RT-qPCR were listed in Table S6. At least three samples in each qPCR analysis were prepared, and three independent experiments were performed.

### Formaldehyde-crosslinked RNA-immunoprecipitation

Cells (1 × 10^7^) seeded in 10-cm plates were cross-linked by 1% formaldehyde at 37 °C for 10 min. Next, 2.5 mol/L glycine was added to the plate at a final concentration of 0.125 mol/L to stop the cross-linking reaction. The cells were washed three times with phosphate-buffered saline (PBS) and scraped from the plate. After centrifugation at 800 ×g for 3 min at 4 °C, the cells were resuspended with 800 μL of RIP buffer (150 mmol/L KCl, 25 mmol/L Tris-HCl pH 7.4, 5 mmol/L EDTA, 0.5 mmol/L DTT, 0.5% NP40, 100 U/mL RNase inhibitor, 100 μmol/L PMSF, 1 μg/mL proteinase Inhibitors) and incubated on ice for 30 min. The cell lysates were centrifugated at 13000 ×g for 10 min; 100 μL of the supernatant were collected as an input control. The remaining lysates were divided into two aliquots and stored at −80 °C until further analysis.

The lysates were incubated with anti-Flag antibody (Sigma-Aldrich) or IgG antibody (Proteintech) overnight at 4 °C. Protein-G agarose beads were washed three times with wash buffer (300 mmol/L KCl, 25 mmol/L Tris-HCl pH 7.4, 5 mmol/L EDTA, 0.5 mmol/L DTT, 0.5% NP40, 100 U/mL RNase inhibitor, 100 µmol/L PMSF, 1 μg/mL proteinase inhibitors) and added to the cell lysate and antibody mixture, and incubated at 4 °C for 2 h. The beads were washed three times with RIP buffer and three times with wash buffer. RNA was extracted using the TRIzol reagent (Invitrogen, Carlsbad, CA, USA) for qRT-PCR.

### Enzyme-linked immunosorbent assay (ELISA)

Huh7 cells were transfected with the HBV 1.1-mer plasmid, pch9-3091, using Lipofectamine 2000 as described above. Cell culture supernatants were harvested 72 h post-transfection. HBsAg and HBeAg were detected using an enzyme-linked immunosorbent assay (ELISA) kit (Kehua Bio-Engineering, Shanghai, China) according to the manufacturer’s instructions. IFN-β was assayed at 24 h post-transfection with a human IFN-β/IFNB ELISA Kit (MM-1641H1, MEIMIAN) according to the manufacturer’s instructions.

### Southern blotting

HBV DNA was extracted from huh7 cells 72 h post-transfection, as described previously [76]. In brief, 5 × 10^6^ cells were lysed with 800 μL HBV DNA lysis buffer (50 mmol/L Tris-HCl pH7.4, 1 mmol/L EDTA, 1%NP-40) on ice for 10 min. The cells were scraped off and centrifugated at 14,000 rpm for 3 min. The supernatants were mixed with 8 μL DNaseI(10 mg/mL) and 8 μL 1 mol/L MgCl_2_, and incubated at 37 °C for 30 min. Then, 40 μL of 0.5 mol/L EDTA was added to stop the reaction. HBV capsids were digested at 55 °C for 2 h after adding 20 μL of 20 mg/mL proteinase K and 80 μL of 10% SDS to cell lysates. Core-associated DNA was extracted using a phenol/chloroform mixture. The extracted DNA was separated on a 1% agarose gel for 4 h at 50 V, followed by denaturation and transfer to Hybound N+ membranes (GE Healthcare) in 20× SSC (3 mol/L NaCl, 0.3 mol/L sodium citrate). The membrane was UV-crosslinked and hybridized with a DIG-labeled probe (DIG HIGH PRIME Kit II). Viral DNA was detected as previously described [77].

### Nuclear and cytoplasmic fractionation

The cells (1 × 10^6^) were harvested 24 h post-transfection. The cytoplasmic and nuclear fractions were extracted using the Cytoplasmic and Nuclear RNA Purification Kit (21000, NORGEN), respectively, according to the manufacturer’s instructions. Purified RNA was reverse-transcribed for qPCR, as described above. U6 and GAPDH serve as internal controls in nucleus and cytoplasm respectively.

### Immuno-precipitation (IP)

IP was performed as previously described [48]. Briefly, cells were washed with cold PBS and then lysed with IP buffer (50 mmol/L Tris-HCl pH 7.5, 1 mmol/L EGTA, 1 mmol/L EDTA, 1% Triton X-100, 150 mmol/L NaCl, 2 mmol/L DTT, 100 μmol/L PMSF, 1 μg/mL proteinase inhibitors) on ice for 30 min. Next, 10% of the cell lysates were collected and used as input control after being centrifuged at 13,000 rpm and 4 °C for 10 min. The remaining lysates were incubated with primary or normal IgG antibodies overnight at 4 °C respectively. Protein-G agarose (catalog number) was added to the mixture and incubated for 1 h at 4, followed by six washes with IP buffer. Immunoprecipitated proteins were eluted in SDS loading buffer. All samples were heat-denatured before SDS-PAGE and western blotting was performed.

### Dual luciferase reporter assay

Huh7 cells were seeded in a 12-well plate and transfected with luciferase vectors using Lipofectamine 2000 (Invitrogen) as described above. Dual-luciferase reporter assays (Promega #E1910) were performed according to the manufacturer’s instructions 24 h post-transfection. Briefly, 100 Μl Passive Lysis Buffer was added to each PBS-washed well. Then, 20 μL of cell lysates was transferred to an Optiplate-96 following a 15 min incubation at room temperature. Firefly and Rellina luciferase activities were assayed using a GloMax Luminometer (Promega). The relative luciferase activity of each sample was calculated as the ratio of firefly to Renilla luciferase activity.

### HBV virus production and infection

HepG2.2.15 cells were cultured for more than three days. The culture supernatants were then harvested and centrifuged at 1000 × *g* and 4 °C for 15 min to remove cell debris. The clarified supernatants was mixed with 5% (w/v) PEG8000 and incubated at 4 °C overnight. The mixture was centrifuged at 4000 rpm and 4 °C for 30 min. The pellets were dissolved in culture medium containing 1% of the starting volume. HepG2-NTCP cells were seeded in 12-well plates prior to HBV infection. The cells were incubated with the collected virus in the presence of 4% PEG8000 and 2% DMSO at 37 °C for 24 h. The cells were washed twice and cultured in fresh medium containing 2% DMSO for more than seven days.

### Ribosome loading

Huh7 cells were cultured and transfected as described above; 2 × 10^7^ cells were treated with 5 mg/mL cycloheximide at 37 °C for 10 min 24 h post-transfection. The cells were then washed three times with PBS and resuspended in 1 mL of ribosome lysis buffer (10 mmol/L Tris-HCl pH 7.4, 5 mmol/L MgCl_2_, 100 mmol/L KCl, 1% Triton X, Protease inhibitor, 2 mmol/L DTT, 100 mg/mL cycloheximide, and RNase inhibitor). RNA was extracted from 10% of the cell lysates and used as the input control. The remaining lysates were loaded into a gradient sucrose solution ranging from 5% to 50% (5% increments) in an SW41 transparent ultracentrifuge tube. The sucrose solution was prepared and stored at 4 °C for 24 h to form a continuous gradient. Sucrose (1 mL) was collected by ultracentrifugation at 30,000 × *g* for 2 h at 4 °C. The OD 260/320 ratio of each fraction was measured using a NanoDrop One. RNA was extracted from the fraction with the highest OD 260/320 nm. The amount of HBV mRNA in this fraction was measured by qPCR.

### RNA oligo pull down

huh7 cells (8 × 10^7^) were harvested and lysed in 2 mL of RIPA buffer (50 mM Tris-HCl, pH 8.0, 0.5 mM EDTA, 0.1% SDS, 1% NP-40, 150 mM NaCl). The cell lysates were centrifuged at 13000 rpm for 15 min at 4 °C. Then, the supernatant was collected as INPUT, and 50 μL of neutravidin beads (29200, Thermo) were washed twice with Tris-buffered saline (TBS). Next, 5 μg of m^5^C modified oligo RNA (Beijing Tsingke Biotech Co., Ltd) or control oligo RNA were added to the beads, the volume was adjusted to 500 μL and rotated at 4 °C for 2 h. The beads were washed three times, and 100 μL of huh7 cell lysate was added to the beads and rotated at 4 °C overnight. The beads were washed again with cold TBS three times, and 40 μL 2x LDS Laemmli buffer was added. Pull-down proteins were analyzed by western blotting.

### RNA stability assay

huh7 cells (1 × 10^4^) were seeded into a 12-well plate. Cells were harvested at 0 h, 2 h, 4 h, 6 h, and 8 h following treatment with 4 μg/mL Actinomycin D (Sigma) at 24 h post-transfection. Total RNA was extracted from huh7 cells at different time points using TRIzol reagent. The same amount of RNA was reverse-transcribed for qPCR, as described above. The proportion of target gene expression relative to 0 h was calculated, and a one-step decay curve was drawn to calculate the half-life.

### Double-stranded DNA oligo pull down

Five micrograms of sense and anti-sense biotin-labeled oligo DNA were incubated at 95 °C for 10 min, and cooled at room temperature. Next, 80 μL of NeutrAvidinTM Agarose (29200, thermo) was washed three times with wash buffer (20 mmol/L Tris-HCl pH 7.5, 100 mmol/L NaCl, 1 mmol/L MgCl2, 0.5 mmol/L EDTA, 0.5 mmol/L DTT). The beads were resuspended in 1 mL of wash buffer, annealed double-stranded oligonucleotide DNAs were added, and the beads were incubated for 2 h at room temperature. The beads were then washed three times with wash buffer and resuspended with 900 μL of pull down buffer (20 mmol/L Tris-HCl pH 7.5, 100 mmol/L NaCl, 1 mmol/L MgCl_2_, 0.5 mmol/L EDTA, 0.5 mmol/L DTT, 4% Glycerol, 10 µg/mL Poly dI-Dc,1× Roche’s protease inhibitor cocktail). Subsequently, 100 μL of cell lysates in RIPA buffer were added to the bead and oligo mixture and incubated overnight at 4 °C. The beads were washed with wash buffer six times and resuspended in 40 μL 2× LDS Laemmli buffer. Proteins were analyzed by western blotting.

### Statistical analysis

Comparisons between two groups were performed using students’ unpaired *t*-tests, while comparisons of the decay curves were performed using two-way ANOVA. *P* ≤ 0.05 is considered statistically significant. All statistical analyses were conducted in GraphPad prism software.

### Supplementary information

Suplementary Imformation

Table S1

Table S2

Table S3

Table S4

Table S5

Original Data File

aj-checklist-CDDIS-23-3260-T

## Data Availability

All data generated or analyzed during this study are included in this published article and its supplementary information files.
