# Supplementary material for: Epigenetic addition of m5C to HBV transcripts promotes viral replication and evasion of innate antiviral responses
Source: Cell Death Dis. 2024 Jan 12;15(1):39. doi: 10.1038/s41419-023-06412-9 (PMC10786922; doi:10.1038/s41419-023-06412-9)
Supplement: Supplementary file 1 — Suplementary Imformation [file 41419_2023_6412_MOESM1_ESM.pdf]

Suplementary Imformation

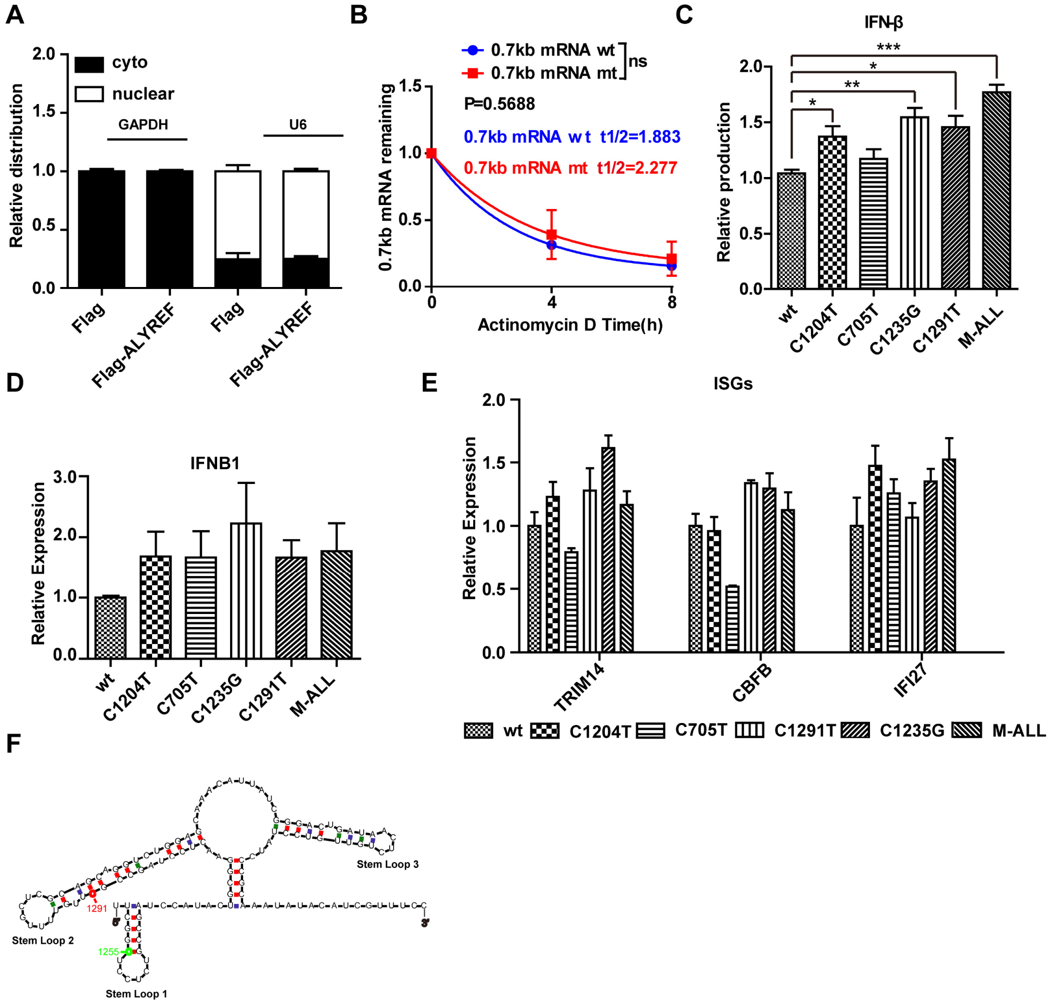

Figure S1 (A) Huh7 cells were transfected with Flag-ALYREF. Subcellular distribution of GAPDH and U6 in cytoplasmic and nuclear fractions examined by qPCR as described in Fig. 2B. (B) The m^{5}C-1291 did not alter the 0.7 kb mRNA stability. Huh7 cells were transfected with cDNA of 0.7 kb mRNA-wt or 0.7 kb mRNA-mutant as described in Fig. 2C. Then, the cells were treated with 4 μg/mL actinomycin D 24 h post-transfection. RNA was harvested and examined by qPCR at 0 h, 4 h or 8 h after treatment. The curve was fitted by one-phase decay in GraphPad Prism. (C) Huh7 cells were co-transfected with poly I:C and HBV 1.1-mer wild type or mutants as described in Fig. 2H. The production of IFN-β was assessed by ELISA at 18h post-transfection. (D, E) Huh7 cells were transfected with wild type or mutant of HBV 1.1-mer, respectively. The expression of IFN-β and ISGs was assessed by RT-qPCR at 18 h post-transfection. (F) RNA secondary structure of HBV 0.7 kb mRNA 5′-UTR predicted by mFold (http://www.mfold.org/mfold/applications/rna-folding-form.php) (Table S2).

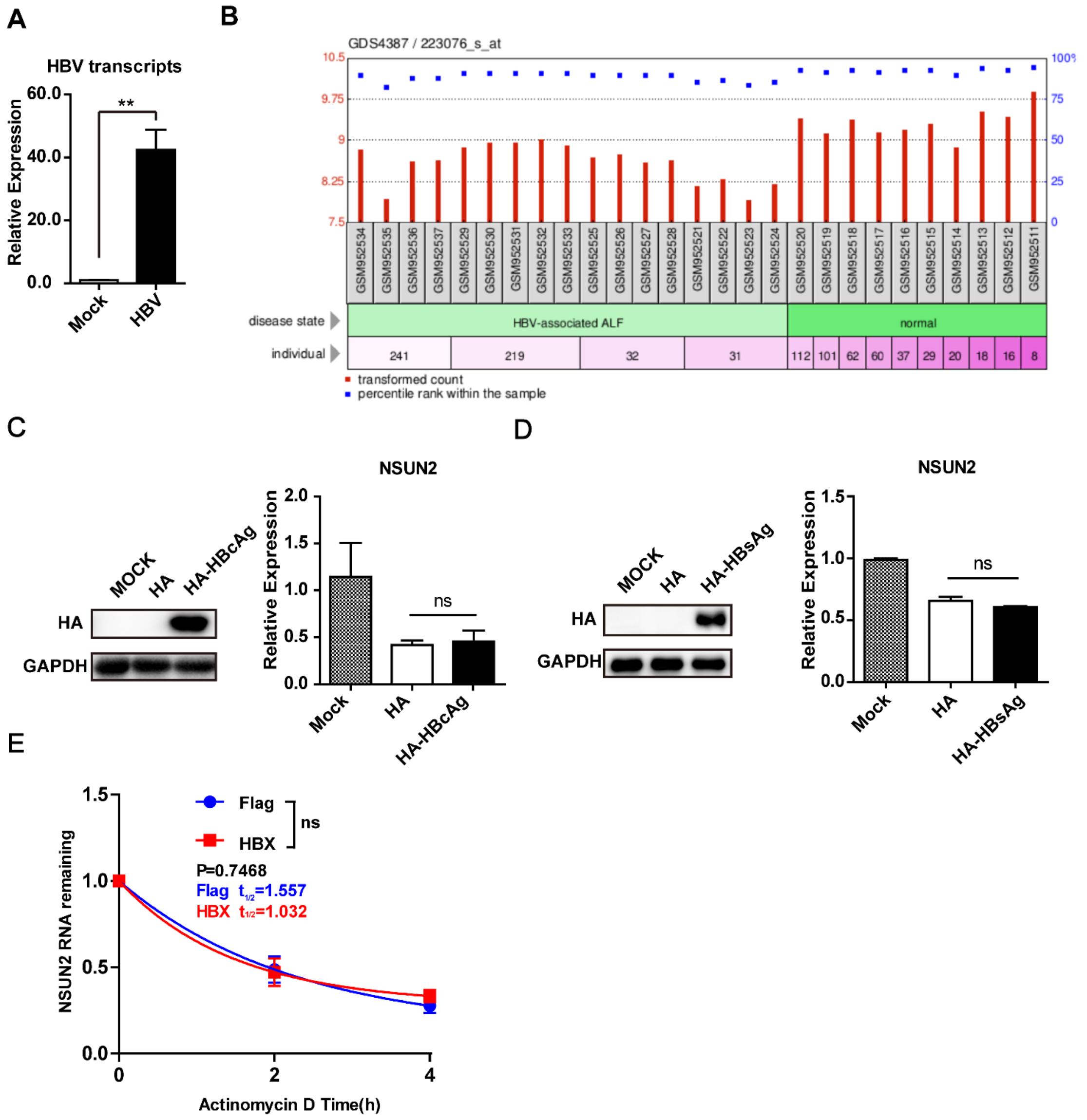

Figure S2 (A) HepG2-NTCP cells infected with HBV as described in Fig. 3A. HBV mRNA level was quantified by qPCR. (B) NSUN2 expressions in HBV-associated ALF patients (https://www.ncbi.nlm.nih.gov/geo/tools/profileGraph.cgi?ID=GDS4387:223076_s_at), from GEO dataset (GSE38941). ALF, acute liver failure. (C, D) HBc and HBs do not alter NSUN2 expression. Huh7 cells transfected with HA-HBc or HA vector (C), or HA-HBs or HA vector (D). Transfection reagent served as the MOCK control. Abundance of HBe or HBs protein accessed by western blot. The RNA level of NSUN2 was quantified by qPCR at 24 h post-transfection. GAPDH was used as an internal control. (E) HBx did not alter the stability of NSNU2 mRNA. Transfection in Huh cells was performed as described in Fig. 3C. Cells were treated with 4 µg/mL actinomycin D at 24 h post-transfection. RNA was harvested and examined by qPCR at 0 h, 2 h or 4 h after treatment. The curve was fitted by one phase decay in GraphPad Prism.

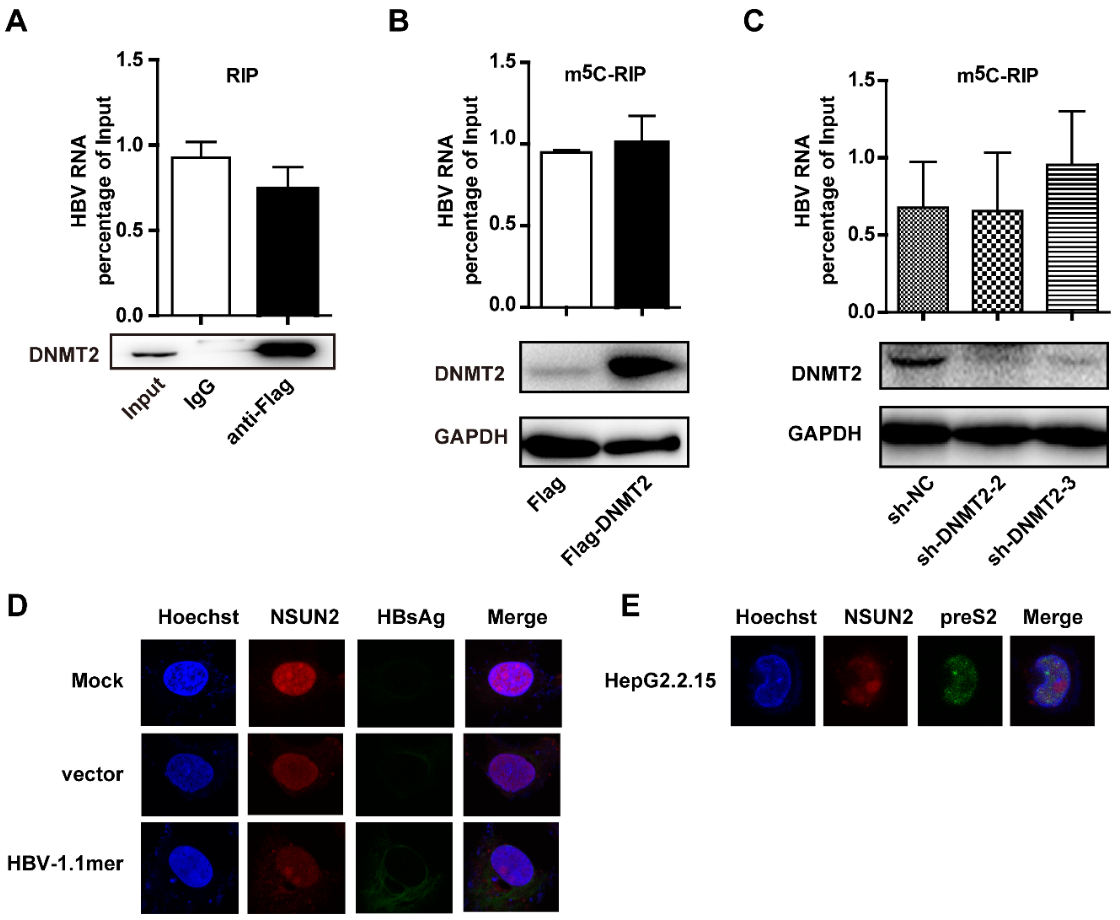

Figure S3 (A–C) DNMT2 does not serve as the methyltransferase of HBV m^{5}C. DNMT2 is not associated with HBV mRNA, verified by RIP assay as described in Fig. 4A (A). Overexpression of DNMT2 (B) or Knock-down of DNMT2 (C) did not alter the total level of m^{5}C in HBV mRNAs determined by m^{5}C-RIP. (D, E) Nuclear localization of NSUN2 is not altered by HBV 1.1-mer transfection (D) or stable HBV ayw strain transfection (E,) determined by immunofluorescence staining. HBs or preS staining indicate efficient HBV transfection (D) or infection (E), respectively.

Table S1-S5

Table S1-S5 are provided in separate excel files.

Table S6

Primers for qPCR assay	sequence(5’ to 3′)
HBV-3′-UTR-F	GTCAACGACCGACCTTG
HBV-3′-UTR-R	TGATTAGGCAGAGGTGAAAAAG
GAPDH-F	GAAGGTGAAGGTCGGAGTC
GAPDH-R	GAAGATGGTGATGGGATTTC
NSUN2-F	GAACTTGCCTGGCACACAAAT
NSUN2-R	TGCTAACAGCTTCTTGACGACTA
U6-F	CTCGCTTCGGCAGCACA
U6-R	AACGCTTCACGAATTTGCGT
IFN-β-F	GCTTGGATTCCTACAAAGAAGCA
IFN-β-R	ATAGATGGTCAATGCGGCGTC
CBFβ-F	AGTTTGATGAGGAGCGAGCC
CBFβ-R	TCTTCTTGCCTCCATTTCCTCC
ISG20-F	TCTACGACACGTCCACTGACA
ISG20-R	CTGTTCTGGATGCTCTTGTGC
IFI6-F	GGTCTGCGATCCTGAATGGG
IFI6-R	TCACTATCGAGATACTTGTGGGT
IFI27-F	TGCTCTCACCTCATCAGCAGT
IFI27-R	CACAACTCCTCCAATCACAACT
TRIM14-F	TGAAGGGGAAATTCACTGAACTC
TRIM14-R	AGCCTCTGGACAGGATCGG
HBx-F	TCTGTGCCTTCTCATCTGC
HBx-R	TCGGTCGTTGACATTGCTG
