# Supplementary figures and images for: Epigenetic addition of m5C to HBV transcripts promotes viral replication and evasion of innate antiviral responses
Source: Cell Death Dis. 2024 Jan 12;15(1):39. doi: 10.1038/s41419-023-06412-9 (PMC10786922; doi:10.1038/s41419-023-06412-9)

**Figure 2**

**A**

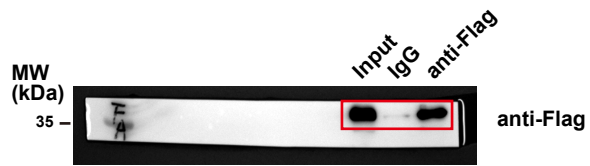

**B**

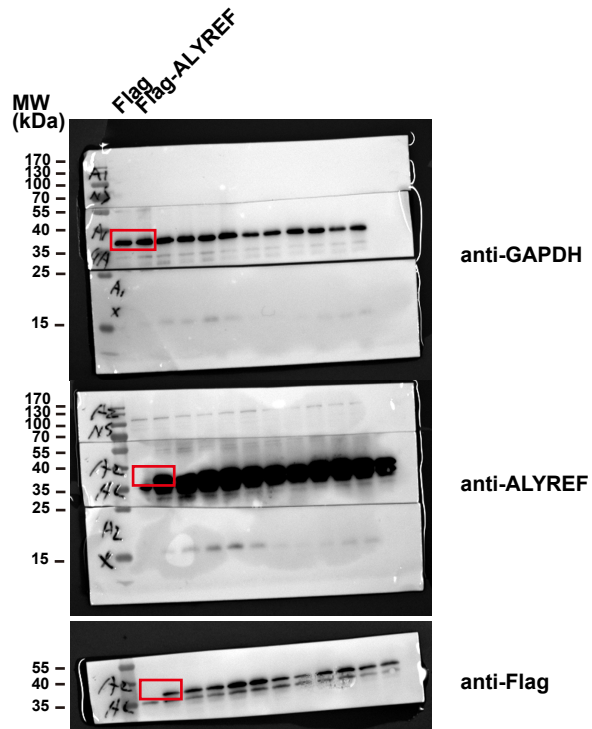

**J**

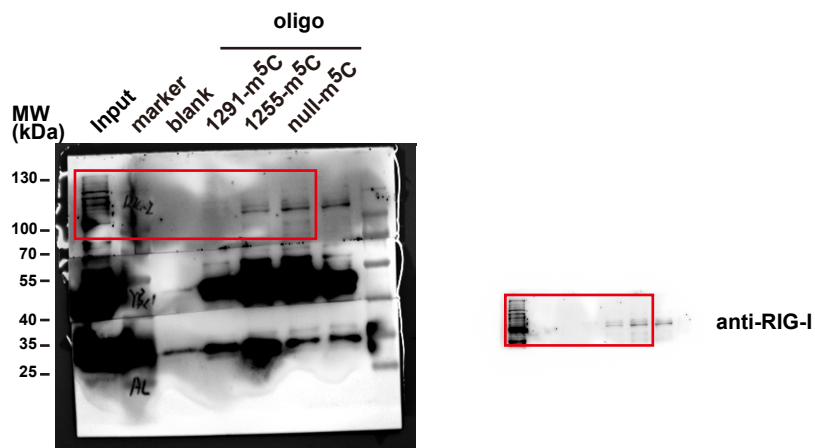

Figure 3

A

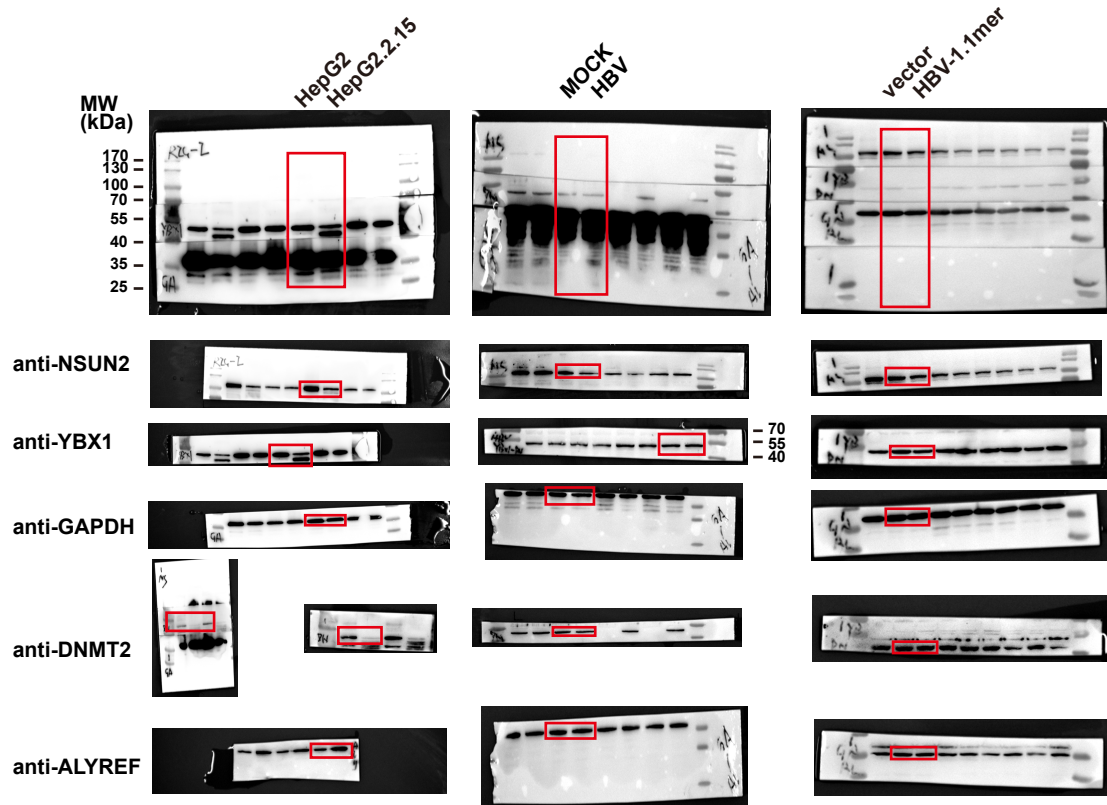

B

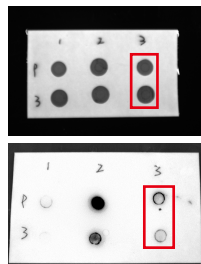

D

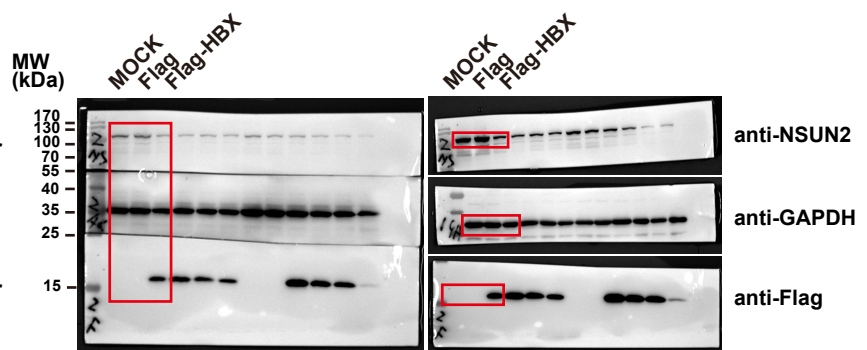

E

| HA-HBX     | + | + | + |
|------------|---|---|---|
| Flag-NSUN2 | + | + | + |

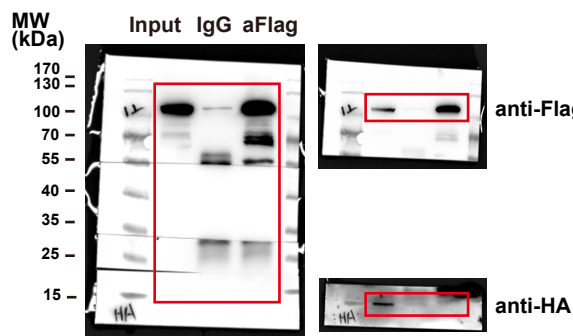

J

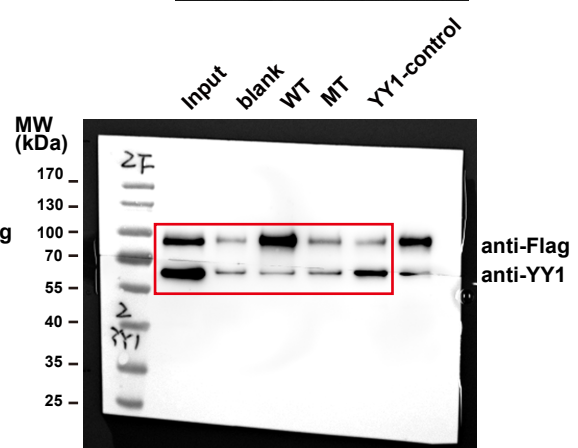

K

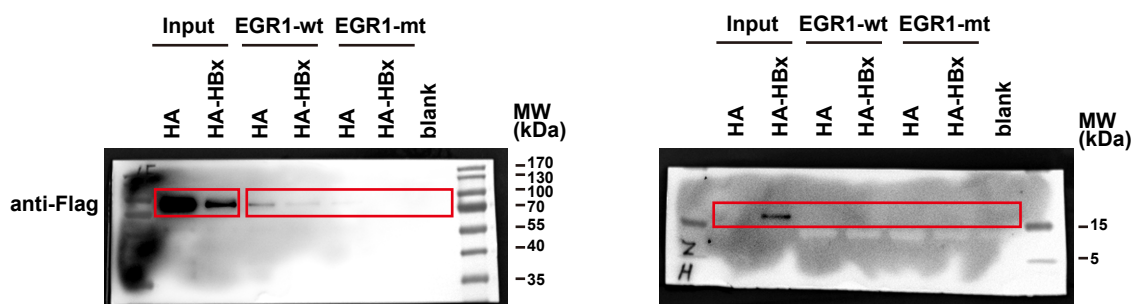

**Figure 4**

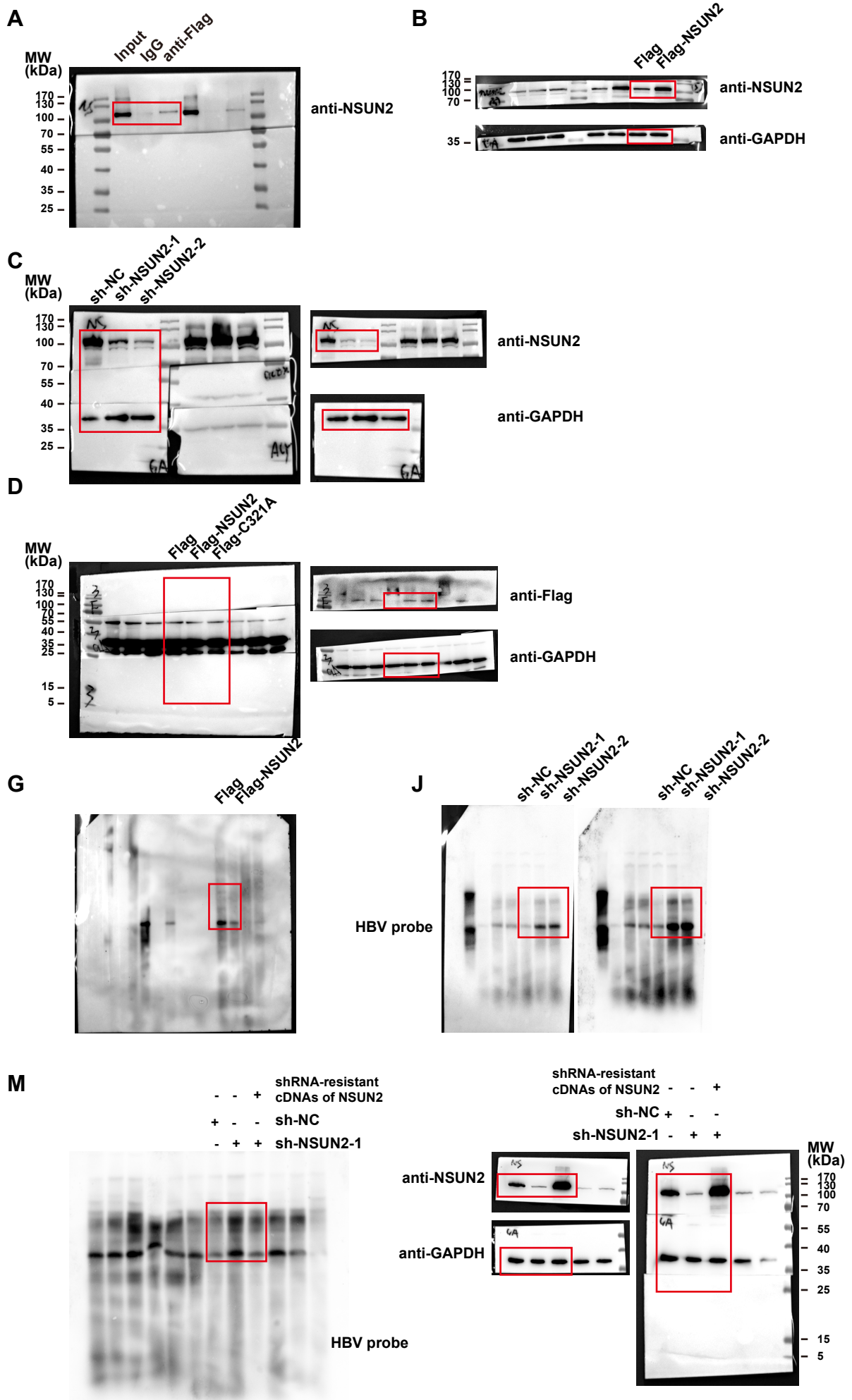

Figure S2

C

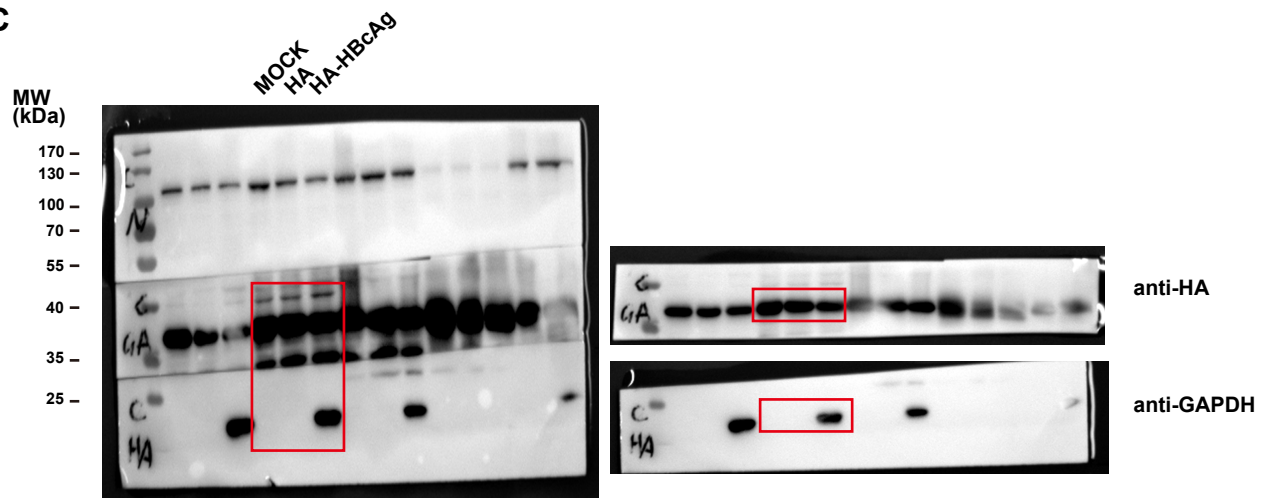

D

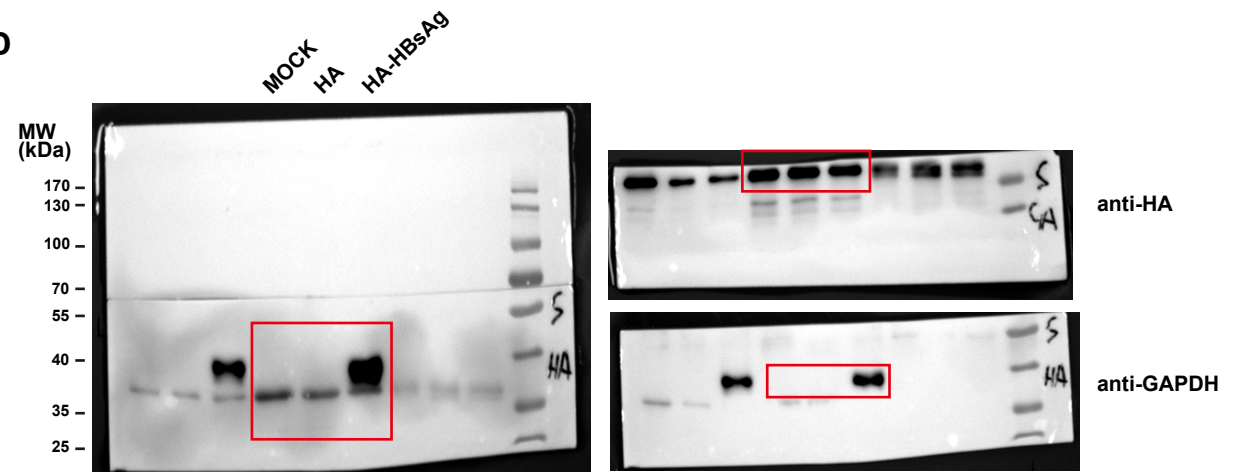

Figure S3

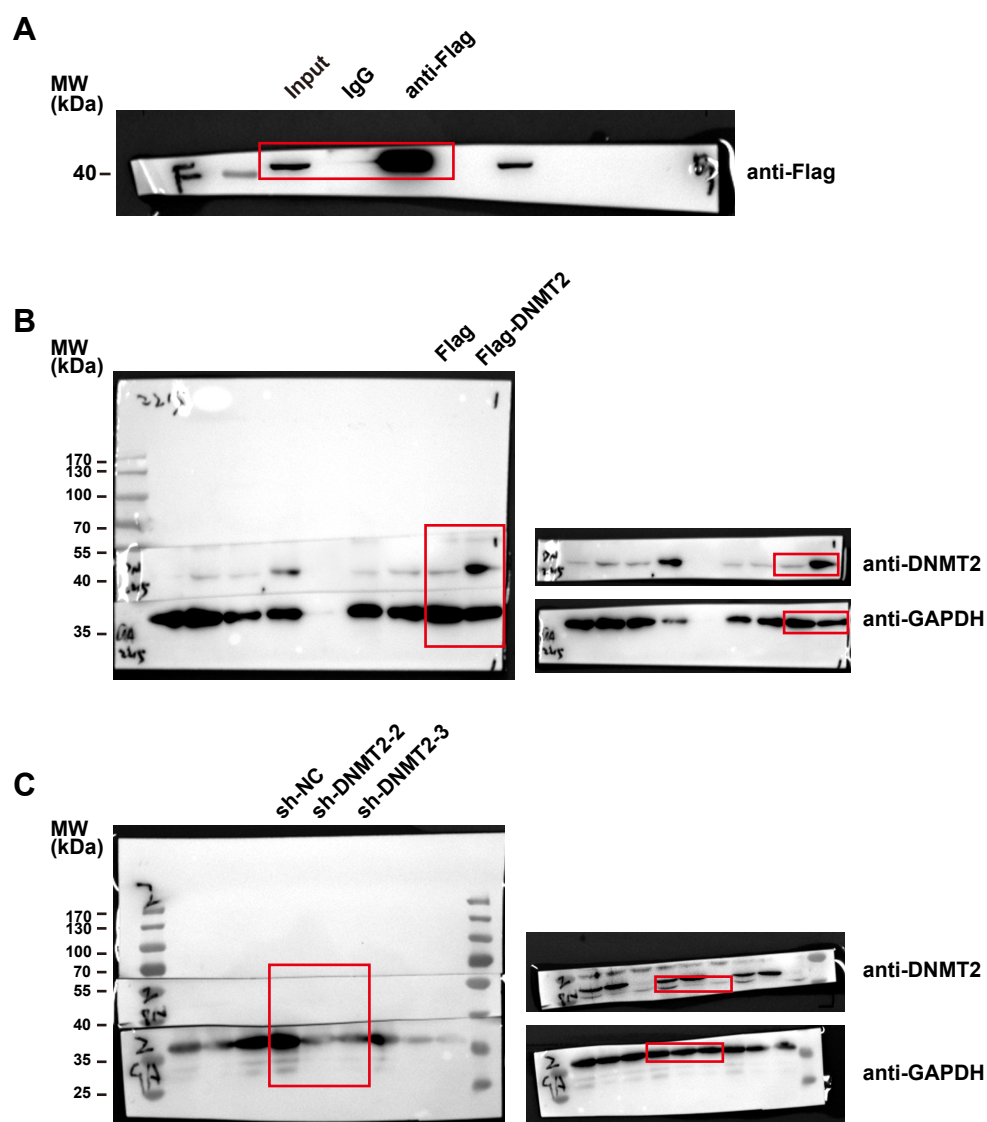

Supplement: Supplementary file 7 — Original Data File [file 41419_2023_6412_MOESM7_ESM.pdf]
